# Fitness costs of resistance to insecticides in insects

**DOI:** 10.3389/fphys.2023.1238111

**Published:** 2023-10-19

**Authors:** Hina Gul, Basana Gowda Gadratagi, Ali Güncan, Saniya Tyagi, Farman Ullah, Nicolas Desneux, Xiaoxia Liu

**Affiliations:** ^1^ MARA Key Laboratory of Pest Monitoring and Green Management, Department of Entomology, College of Plant Protection, China Agricultural University, Beijing, China; ^2^ Division of Crop Protection, ICAR-National Rice Research Institute, Cuttack, Odisha, India; ^3^ Department of Plant Protection, Faculty of Agriculture, Ordu University, Ordu, Türkiye; ^4^ Department of Entomology, BRD PG College, Deoria, Uttar Pradesh, India; ^5^ State Key Laboratory for Managing Biotic and Chemical Threats to the Quality and Safety of Agro-products, Institute of Plant Protection and Microbiology, Zhejiang Academy of Agricultural Sciences, Hangzhou, China; ^6^ Université Côte d'Azur, INRAE, CNRS, UMR, ISA, Nice, France

**Keywords:** integrated pest management, selection pressure, ecotoxicology, toxins, fitness costs, life table, biological traits

## Abstract

The chemical application is considered one of the most crucial methods for controlling insect pests, especially in intensive farming practices. Owing to the chemical application, insect pests are exposed to toxic chemical insecticides along with other stress factors in the environment. Insects require energy and resources for survival and adaptation to cope with these conditions. Also, insects use behavioral, physiological, and genetic mechanisms to combat stressors, like new environments, which may include chemicals insecticides. Sometimes, the continuous selection pressure of insecticides is metabolically costly, which leads to resistance development through constitutive upregulation of detoxification genes and/or target-site mutations. These actions are costly and can potentially affect the biological traits, including development and reproduction parameters and other key variables that ultimately affect the overall fitness of insects. This review synthesizes published in-depth information on fitness costs induced by insecticide resistance in insect pests in the past decade. It thereby highlights the insecticides resistant to insect populations that might help design integrated pest management (IPM) programs for controlling the spread of resistant populations.

## 1 Introduction

Insect pests cause severe agricultural damage, leading to high financial and environmental costs worldwide. Despite multiple possible alternative pest management methods (Lu et al., 2012; Gurr et al., 2017; Jactel et al., 2019; Verheggen et al., 2022), chemical pesticides are usually the most commonly used method to control insect pests (Deguine et al., 2021). However, under continuous selection pressure of chemical insecticides, insects have developed resistance against different groups of insecticides (Koo et al., 2014; Ma et al., 2019; Ullah et al., 2020a; Pires et al., 2021; Li et al., 2022a). Fitness is the quantitative representation of an organism reproductive success. Fitness costs are a trade-off between biological traits in which alleles confer higher fitness in one condition, such as selection pressure to insecticides, while reduced fitness in another condition, such as without insecticide selection (Ullah et al., 2020b; Singarayan et al., 2021).

Fitness costs related to insecticide resistance occur in insects when the development of insecticide resistance is accompanied by energy costs or significant physiological disadvantages that affect the fitness of the target insects compared to their susceptible counterparts in the population (Kliot and Ghanim, 2012). Fitness costs are linked not only with insecticide resistance development but also with several other phenomena. Fitness costs are associated when insects adapt to new habitats, combat different stressors, and adapt to toxic secondary metabolites of new host plants (Kliot and Ghanim, 2012). The most common mechanisms of insecticide resistance included 1) metabolic detoxification due to the expression of metabolic genes, 2) target site mutations, 3) decreased penetration/increased excretion, and 4) behavioral resistance. The upregulation of detoxification genes is linked with insecticide resistance, resulting in fitness costs following the resource and energy reallocation due to the expense of metabolic and developmental processes (Grigoraki et al., 2017).

**FIGURE 1:**
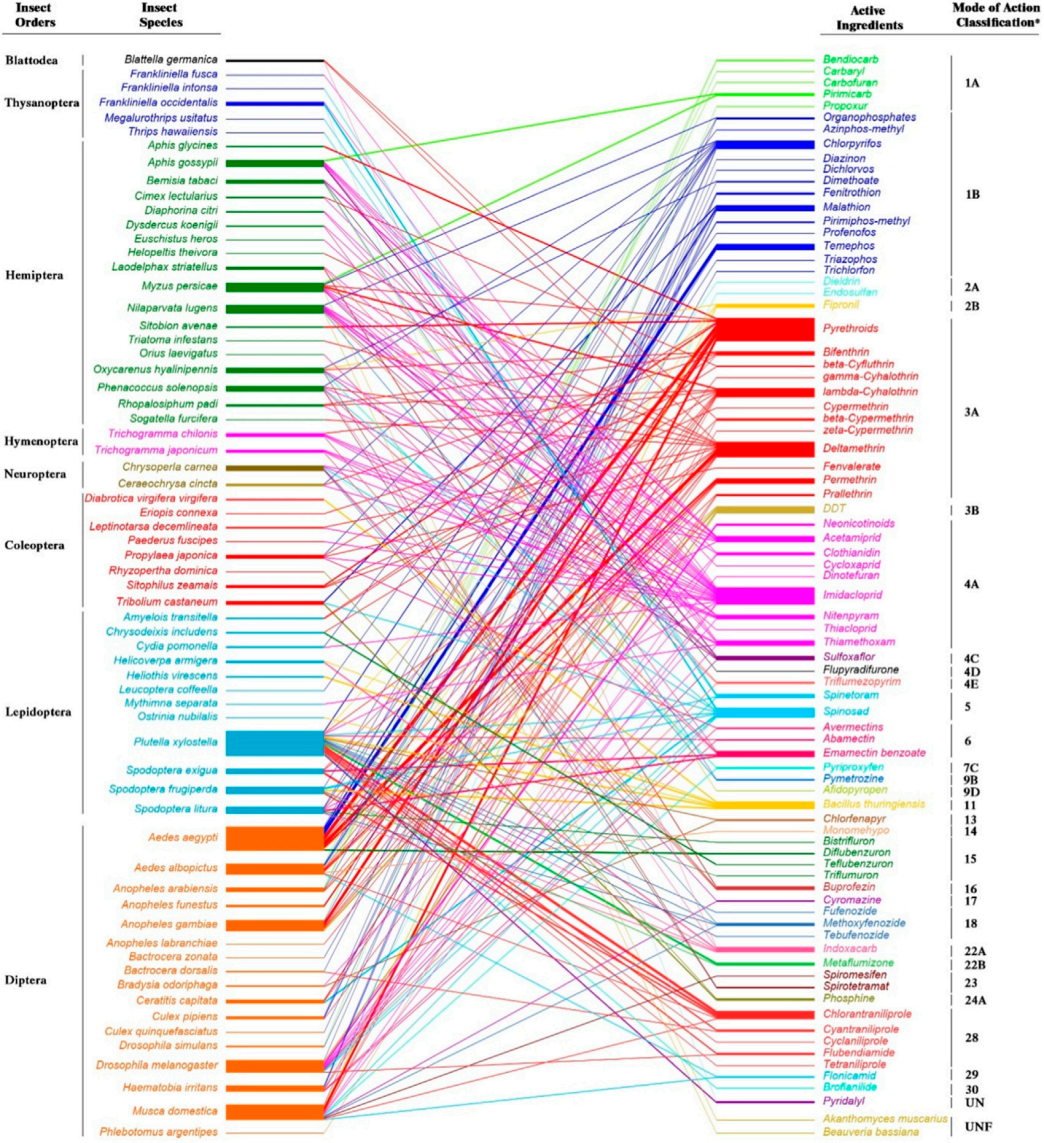
Studies about fitness costs of insect pest species associated with different insecticides with Mode of Action Classification. The width of the bars denote the number of studies in the literature of the last decade reviewed from Science Citation Index Expanded within the Clarivate Web of Science database (https://www.webofscience.com) using “insecticide”, “fitness”, and “resistance” as keywords. *According to the Insecticide Resistance Action Committee (IRAC, https://irac-online.org/, Edition: 10.5, March 2023) Mode of Action Classification Groups: 1: Acetylcholinesterase (AChE) inhibitors; 1A Carbamates, 1B Organophosphates. 2: GABA-gated chloride channel blockers; 2A Cyclodieneorganochlorines, 2B Phenylpyrazoles. 3: Sodium channel modulators; 3A Pyrethroids, 3B DDT, Methoxychlor. 4: Nicotinic acetylcholine receptor (nAChR) competitive modulators; 4A Neonicotinoids, 4C Sulfoximines, 4D Butenolides, 4E Mesoionics. 5: Nicotinic acetylcholinereceptor(nAChR) allostericmodulators–Site I; Spinosyns. 6: Glutamate-gatedchloride channel (GluCl) allostericmodulators; Avermectins, Milbemycins. 7: Juvenile hormone mimics; 7C Pyriproxyfen. 9: Chordotonal organ TRPV channel modulators; 9B Pyridine azomethine derivatives, 9D Pyropenes. 11: Microbial disruptors of insect midgut membranes; *11 Bacillus thuringiensis.* 13: Uncouplers of oxidative phosphorylation via disruption of the proton gradient; Pyrroles, Dinitrophenols, Sulfluramid. 14: Nicotinic acetylcholine receptor (nAChR) channel blockers; Nereistoxin analogues. 15: Inhibitors of chitin biosynthesis affecting CHS1; Benzoylureas. 16: Inhibitors of chitin biosynthesis, type 1; Buprofezin. 17: Moulting disruptors, Dipteran; Cyromazine. 18: Ecdysone receptor agonists; Diacylhydrazines. 22: Voltage-dependent sodium channel blockers; 22A Oxadiazines, 22B Semicarbazones. 23: Inhibitors of acetyl CoA carboxylase; Tetronic and Tetramicacid derivatives. 24: Mitochondrial complex IV electron transport inhibitors; 24A Phosphides. 28: Ryanodine receptor modulators; Diamides. 29: Chordotonal organ nicotinamidase inhibitors; Flonicamid. 30: GABA-gated chloride channel allosteric modulators; Meta-diamides, Isoxazolines. UN: Compounds of unknown or uncertain MoA; Pyridalyl. UNF: Fungal agents.

The genes responsible for resistance may be of homozygous (RR) or heterozygous (Rr) genotype. It is essential to know the genetic background of the resistance genes to study the fitness costs attached to it. Since heterozygotes may be relatively prevalent in the early phases of pesticide selection, the fitness costs in heterozygote-resistant strains are more important than in the homozygote-resistant population. It is necessary to conduct further research on the dominance of any putative pleiotropic effects of resistance in these heterozygous individuals [for details, check these key documents 15,16]. Under certain conditions, fitness costs in an insecticide-resistant insect pest population showed reduced survival and reproduction and slowed the evolution of resistance (Ullah et al., 2020c). The fitness of insecticide-resistant populations is impacted by the development of insecticide resistance, which is often associated with a high energetic cost (Grigoraki et al., 2017). Several studies reported fitness costs associated with different classes of insecticide resistance on insect pest species such as *Thrips hawaiiensis*, *Aphis gossypii*, *Nilaparvata lugens*, *Plutella xylostella*, *Spodoptera* spp*., Aedes* spp*., Bradysia odoriphaga*, and *Musca domestica* (Abbas et al., 2016a; Steinbach et al., 2017; Zhang et al., 2018a; Fu et al., 2018; Ullah et al., 2020c; Shan et al., 2021; Ullah et al., 2021) (Figure 1). To better understand the manifestation of these fitness costs and the scope of this phenomena, Freeman et al. (2021) did a detailed review of literature on studies that examined the fitness costs of pesticide resistance and examined each class of insecticide individually and collectively. In 60% of the trials, pesticide resistance has a cost, especially for reversion of resistance and reproduction measurements, according to more than 170 papers on the fitness costs of insecticide resistance. There were variances among insecticide classes, with fitness costs being more seldom observed for organochlorines.

Our current review presents the body of literature on fitness costs due to insecticide resistance in a few important insect orders along with fitness advantage over a decade. Though a few reviews have been published on fitness cost associated with insecticide resistance, they are mostly based on the class of insecticides (Bass, 2017; Freeman et al., 2021). In-depth knowledge about the fitness costs induced by any insecticide might help design an integrated pest management (IPM) program to control the spread of a resistant population of insect pests. In this review, we have structured our discussion through insect orders to explore the fitness costs induced by various class of insecticides based on studies conducted over the past decade.

## 2 Blattodea

Blattodea contains about 3,500 to 4,000 species of cockroaches identified, which can be divided into five families: Cryptocercidae, Blattidae, Blattellidae, Blaberidae, and Polyphagidae. The most important pest specie was the German cockroach, *Blattella germanica*. Its extraordinary ability to acquire resistance to harmful insecticides was a prime example of adaptive evolution. The most frequent approach to control *B. germanica* in homes, flats, and commercial kitchens was to apply insecticides (Dingha et al., 2013; Wang et al., 2019a). *Blattella germanica* transmits several microorganisms (Lee et al., 2021), causes allergic reactions (Eggleston, 2017; Wang et al., 2020a; Lee et al., 2021), and poses a health risk because it serves as a mechanical vector for a variety of harmful bacteria (Brenner and Kramer, 2019). Many factors, including physiological resistance to the insecticide, cross-resistance (Abbas and Shad, 2015), and contamination of the insecticide deposit, can affect the effectiveness of insecticides used to control *B. germanica* (Lee et al., 2022). During resistance onset, the fitness cost may be high, but as resistance advances with constant selection pressure, these costs may be reduced or eliminated due to the replacement of high energetic resistance alleles with lesser ones or due to the selection of modifier alleles that reduce the fitness costs (Basit, 2019).

Insects with resistant genotypes pay an energetic cost that reduces their fitness compared to susceptible conspecifics. Hemiptera, Diptera, Coleoptera, and Lepidoptera have all been observed with this trait (Kliot and Ghanim, 2012; Mengoni and Alzogaray, 2018; Castellanos et al., 2019). Compared to other resistant strains of German cockroach, certain pyrethrin- and allethrin-resistant cockroaches have an irregular pattern of development among the nymphs and a lower total fecundity (Zhang and Yang, 2019). Insecticide-resistance is typically connected with life history costs that prevent it from being fixed. Fitness-related costs had delayed developmental stages and shorter adult lifespan (Hardstone et al., 2014). Experimental conditions, including feeding, relative humidity and temperature, strain origin, aggregation effects, and pesticide category, may influence cost variations (Zhang et al., 2017; Wang et al., 2020b; Chong et al., 2022). Jensen et al. (2016) showed that indoxacarb-selected cockroaches had poorer survival to maturity, decreased adult body size, and prolonged development time with reinforcing interactions, showing that poor nutritional condition might increase the cost of pesticide adaptation and fitness costs via interactions with insecticide resistance.

## 3 Thysanoptera

Like other insect orders, insecticide resistance among thrips species poses a significant challenge to effective pest management strategies. Understanding the interplay between insecticide resistance and associated fitness costs is critical. The studies show case diverse insights into the dynamics of insecticide resistance. The study by Nakao et al. (2014) investigated the developmental and ovipositional behaviors of pyrethroid-resistant and pyrethroid-susceptible strains of *Thrips tabaci* (Thysanoptera: Thripidae), on different persimmon and green bean varieties. Despite differences in pyrethroid susceptibility, the pyrethroid-resistant strains had lower fecundity than susceptible strains on green bean leaves, suggesting that resistance did not substantially affect other aspects of pest behavior in commercial persimmon orchards. Gao et al. (2014) enrich the discourse by unraveling cross-resistance patterns and the biochemical mechanisms driving resistance to thiamethoxam in *F. occidentalis* (Thysanoptera: Thripidae), displaying low survival in first instar larvae, pupation percentage and fecundity in resistant ones. Li et al. (2017) reveal that spinosad resistance can lead to decreased fecundity and reduced body size and affect the feeding behaviors of *F. occidentalis*. Wan et al. (2021), delve into the intricate relationship between spinosad resistance and transmission efficiency of Tomato spotted wilt orthotospovirus (TSWV) by *F. occidentalis*, further underlining the potential impacts of resistance on vector competence and concluded that spinosad resistance in *F. occidentalis* reduced pupation rate, sex ratio and male longevity and increased vector competence. On the other hand, the influence of host plants emerges as a crucial factor in thrips resistance dynamics. Li et al. (2022b) highlight how spinetoram resistance may alter the host preference of *F. occidentalis*, potentially driven by shifts in detoxification enzyme activities. In addition, cost of spinetoram resistance leads shorter preadult and adult longevity in tested hosts, i.e., broader bean and eggplant. Fu et al. (2018), highlights the rapid increase in resistance to spinetoram in *Thrips hawaiiensis* (Thysanoptera: Thripidae), accompanied by a decline in resistance ratios over generations. Similarly, Hua et al. (2023), demonstrate how spinosad resistance in *F. occidentalis* (Thysanoptera: Thripidae), incurs fitness costs, affecting fecundity and ovary development. Furthermore, the complexity of resistance in pest management strategies is evident in the study by Chappell et al. (2019), which demonstrates that fitness costs of imidacloprid resistance in *Frankliniella fusca* (Thysanoptera: Thripidae), may not necessarily impede resistance evolution alone, as consistent insecticide use can offset these costs. The study by Wang et al. (2020c) determined that the *F. occidentalis*, exhibited high susceptibility to the insecticide pyridalyl in field populations from 2016 to 2017 in China. However, a laboratory-selected pyridalyl-resistant strain showed no cross-resistance to other insecticides, and its resistance was inhibited by piperonyl butoxide and diethyl maleate. The pyridaly resistant *F. occidentalis* strain had lower pupation and emergence rates and reduced female fecundity, indicating resistance-related fitness costs. The study by Fu et al. (2022) found that the extensive use of spinetoram in controlling two closely related thrips, *Megalurothrips usitatus* and *F. intonsa* (Thysanoptera: Thripidae), led to the displacement of *F. intonsa* by *M. usitatus* on cowpea crops due to interspecific competition. Exposure to spinetoram favored *M. usitatus* dominance, and the development of higher resistance to spinetoram in *M. usitatus* compared to *F. intonsa* suggests a connection between resistance evolution and competitive interactions. After laboratory selection, both species showed increased resistance, with *M. usitatus* having higher resistance and no associated fitness costs, potentially explaining the recent dominance shift. In contrast, spinetoram resistant *F. intonsa* showed a lower net reproduction rate, intrinsic rate of increase and finite rate of increase.

By synthesizing insights from these diverse studies, the complicated relationships between resistance, fitness costs, and host interactions are paramount in devising effective pest management strategies against thrips pest species.

## 4 Hemiptera

Hemipterans suck the sap from plants’ vascular system, which can lead to the yellowing, drying and wilting of plants. Additionally, in the case of planthoppers and leafhoppers, a more severe symptom known as “hopper burn” can be observed if the extent of damage is high. These insects also serve as vectors for several plant diseases, enabling their control as a critical component in disease management (Shi et al., 2014). Hemipterans, such as aphids, whiteflies, and plantoppers, are generally of small size with a short life cycle and high fecundity, which enable the use of insecticide as the most feasible option for their control. However, more frequent use of insecticides develops resistance in hemipterans against them with varying effects on their fitness traits. Resistance build-up and associated fitness trade-offs in insects have been extensively studied individually for various insecticidal groups, such as organophosphates, carbamates, pyrethroids, and neonicotinoids (Saeed et al., 2021; Ullah et al., 2022; Valmorbida et al., 2022; Walsh et al., 2022). The common finding in all of the above studies is that after cessation of selection pressure of insecticides, which leads to the reversion of resistance, the earlier resistant insect strains could compensate for the reduced biological fitness in more or less time. Likewise, recently, such studies have also been documented for combination insecticides. In a study on cabbage aphid, *Brevicoryne brassicae* in Iran, exposure to multiple sublethal doses of thiamethoxam-lambda cyhalothrin, a combination of a neonicotinoid and a pyrethroid, had shown an adverse effect on their offsprings’ reproductive rate, survival rate, and fecundity. This could be due to increased toxicity, leading to high selection pressure on insects, which was apparent only from the first generation (Mahmoodi et al., 2020). One of the most devastating hemipterans, i.e., whitefly, *Bemisia tabaci*, showed slow development, low survival, and reduced egg laying due to its resistance to acetamiprid (Roy et al., 2019). The extended nymphal period was also reported due to resistance to pyriproxyfen, an insect growth regulator (Singh and Chandi, 2019). In Korea, there is a prevalence of genetic displacement of cluster 2 over 1 in *B. tabaci* populations primarily based on its high resistance levels to thiamethoxam compared to cluster 1. Higher LC_50_ values of the cluster 2 population for thiamethoxam are due to the metabolic factor rendered by elevated cytochrome P450 activity (Park et al., 2021).

The brown planthopper, *N. lugens*, is the most significant insect pest in the world’s main rice-growing areas. It has gained resistance to many insecticide classes over time (Malathi et al., 2017). A recent example is its resistance to triflumezopyrim, which inhibits the nicotinic acetylcholine receptor and belongs to a new family of mesoionic insecticides (Liao et al., 2021; Qin et al., 2021). All the important biological parameters, such as lifespan, female adult period, fecundity, and hatchability, were decreased in the resistant strain compared to the susceptible strain, resulting in a relative fitness of 0.62 (Qin et al., 2021). The pre-adult time and total pre-oviposition duration were increased. Additionally, there was a general decline in the resistant population (Qin et al., 2021). Similar patterns of decline in the biological fitness of *N. lugens* strains resistant to imidacloprid (Sanada-Morimura et al., 2019), nitenpyram (Zhang et al., 2018a), sulfoxaflor (Liao et al., 2019), and clothianidin (Jin et al., 2021) were also observed. The reduced fitness of insects in the presence of insecticides will slow down resistance build-up. However, in the case of hemipterans, their high fecundity and shorter life cycle will aid in developing resistance quickly and presumably regain their full health potential rapidly in the insecticide-free environment (Qin et al., 2021).

## 5 Hymenoptera

As pesticides are an integral part of the crop ecosystem to manage agricultural pests and diseases, they can have a variety of direct (lethal) and indirect (sublethal) effects on target and non-target organisms (Ndakidemi et al., 2016). The adverse consequences of insecticides on natural enemies are frequently cited as a major barrier to implementing Integrated Pest Management (IPM) programs (Ndakidemi et al., 2016). Although pesticide resistance in beneficial insects was less understood, it has been established in several species (Biondi et al., 2015; Sparks and Nauen, 2015; Lommen et al., 2017; Overton et al., 2021; Schmidt-Jeffris et al., 2021; Tanda et al., 2022). Natural enemies are more vulnerable to pesticides than herbivorous insect pests (Bielza et al., 2020). Parasitic Hymenoptera is less likely to develop resistance compared to other insects (Bielza et al., 2016). *Trichogramma* spp. egg parasitoids are among the most important biological control agents of Lepidoptera pests globally and enable controlling insect pests before they damage plants (Sithanantham et al., 2013; El-Arnaouty et al., 2014; Huang et al., 2020; Zang et al., 2021; Zhang et al., 2021). The use of *Trichogramma* wasps has been developed through extensive applied research (Wang et al., 2014; Du et al., 2018; Hou et al., 2018; Wang et al., 2019b; Gontijo et al., 2019; Guo et al., 2019; Wang et al., 2021; Zhang et al., 2021), their use being mostly through artificial inundative releases (El-Arnaouty et al., 2014; Huang et al., 2020; Zang et al., 2021), although natural parasitism has been reported for possible conservation biological control (Biondi et al., 2013; Bagheri et al., 2019; Salas Gervassio et al., 2019). They are used for biological control of many insect pests on various crops, e.g., vegetables and tree crops, and stored products (Chailleux et al., 2013; Marchioro et al., 2015; Khan and Ruberson, 2017; Gontijo et al., 2019; Qu et al., 2020; Zang et al., 2021).

Based on numerous laboratory and field investigations, most studies have focused on sublethal effects rather than examining the fitness costs of insecticides in Hymenopteran insects. In the study by Xie et al. (2022) the researchers aimed to develop insecticide-tolerant strains of *Trichogramma* parasitoids, which are used as biocontrol agents against lepidopteran rice pests in rice fields. They exposed *Trichogramma japonicum* and *Trichogramma chilonis* to sublethal doses of imidacloprid, thiamethoxam, buprofezin, and nitenpyram targeting rice planthoppers in a laboratory setting. *Trichogramma japonicum* showed the highest increase in tolerance to imidacloprid after successive treatments, and *T. chilonis* also displayed increased tolerance to these insecticides. Over time, the emergence and deformity rates of the treated *Trichogramma* species gradually recovered, and the fecundity of *T. japonicum* treated with thiamethoxam was significantly higher compared to the control, while *T. chilonis* treated with certain insecticides exhibited differences in fecundity. The study suggests that developing insecticide-tolerant *Trichogramma* strains, especially pairing *T. japonicum* with imidacloprid, could enhance the integration of biological control methods with traditional chemical strategies in integrated pest management for rice agroecosystems.

*Trichogramma* spp. wasps are particularly vulnerable to most broad-spectrum insecticides (Cheng et al., 2018). As a result, pesticides and *Trichogramma* spp. have traditionally been thought to be incompatible as pesticides negatively impact the parasitoids’ fitness in agroecosystems. An example of this scenario was documented by Tabebordbar et al. (2020) that Metasystox, dichlorodiphenyltrichloroethane (DDT), and Metasystox negatively affected *Trichogramma evanescens* by decreasing longevity and fecundity, as well as increasing adult mortality after their emergence.

Several novel insecticides, e.g., methoxyfenozide, tebufenozide and spinosad, have been developed and tested against lepidopteran pests in cotton to overcome insecticide resistance, support natural enemies, and reduce health concerns (Sifakis et al., 2017). The effect of certain pesticides on emergence appears to be associated with the preimaginal development stage at the time of exposure (Amandeep et al., 2012; Khan and Ruberson, 2017).

The impact of resistant pest populations on natural enemies in the action of pesticides has yet to be determined. The interference identified in the performance of *T. pretiosum* emphasizes the necessity of understanding how improper insecticide use can harm the fitness of the natural enemy. On the other hand, *T. pretiosum* can still be utilized in IPM management programs since it maintains its effectiveness even in the presence of resistant populations in the field whenever host eggs of *S. frugiperda* populations had some resistance when over six generations of exposure to metaflumizone, the biological activity of *T. pretiosum* was decreased (Barbosa et al., 2021). It was discovered that eggs originating from the resistant population had lower parasitism percentages, emergence, and parasitoids/eggs, but females from the susceptible population survived longer than those from the resistant population. However, neither the sex ratio nor the male longevity was altered.

Pesticide resistance results from mutations that replace alleles, and the selection pressure (Van Leeuwen et al., 2020) enhances the genotypic diversity of the original population. Due to its intricacy, the co-evolutionary process that combines parasitoids and hosts in the field (Martinez et al., 2014) cannot be examined in laboratory settings. Consequently, the interference in the examined biological parameters was at its greatest. When the host may acquire physiological resistance, the parasitoids evolve tactics to combat this resistance (George et al., 2021).

*Cotesia plutellae* was a prominent natural enemy of the diamondback moth. *P. xylostella* had developed extensive resistance to various insecticides, including microbiological Bt formulations, and had been a devastating pest of brassica crops (Sparks et al., 2012; Li et al., 2016; Lin et al., 2020; Banazeer et al., 2021). In laboratory investigations, the effects of Bt plants on a larval endoparasitoid of both Bt-susceptible and Bt-resistant *P. xylostella* strains were compared to the deadly effects of Cry1Ac-expressing transgenic oilseed rape (*Brassica napus*) on the endoparasitoid, *Cotesia plutellae*. Resistant *P. xylostella* larvae feeding on highly resistant Bt plants were ideal hosts for *C. plutellae* growth.

The resistance mechanisms in aphids have been linked to fitness costs that are both inhibitory and pleiotropic. But sometimes, parasitoids can take advantage of trade-offs in the insecticide-resistant clone to counteract insecticide resistance. For example, the parasitization (mummification) rate of *Aphidius ervi* towards the insecticide-resistant (knockdown resistance (kdr) Sitobion avenae) clones was noticeably higher than their insecticide-susceptible counterparts (Sitobion avenae). This might be due to the smaller heterozygous (kdr-SR) clones’ ineffective warding behavior (Jackson et al., 2020). This is consistent with Onstad and Flexner (2023), who discovered that insecticide-resistant peach potato aphids (*Myzus persicae*) were mummified at a higher rate than their insecticide susceptible counterparts. When selected for by a higher trophic level, pesticide resistance genes sometimes cause maladaptive behaviors that have a detrimental pleiotropic effect on fitness. For example, eight parthenogenetic *M*. *persicae* clones (representing various pesticide resistance genotypes) were subjected to alarm pheromones to confirm the degree of response. The clones were then exposed to adult female parasitoids, *Diaeretiella rapae,* both in the presence and absence of measured amounts of alarm pheromone. Results exhibited that clones with a consistently high alarm response (insecticide-susceptible forms) when compared to aphids with a low alarm response (insecticide-resistant forms), displayed a variety of behaviors during and after parasitoid attack that were significantly associated with greater survival (avoidance of parasitism) (Onstad and Flexner, 2023).

## 6 Neuroptera

The detrimental consequences of insecticide use on natural enemies have been a significant focal point within pest management science. Lacewings stand out as crucial natural predators because of their capability to control a wide range of pests and are naturally distributed extensively across various agricultural production areas. Using insecticide-resistant natural enemies can potentially suppress pests in numerous cropping systems where chemical pest management is a prevalent approach. The primary focus of insecticide resistance research has been directed towards common green lacewing, *Chrysoperla carnea* (Neuroptera: Chrysopidae).

For example, a study by Mansoor et al. (2013) involved selecting a field-collected population of the common green lacewing *C. carnea* for resistance to the insecticide emamectin benzoate in a laboratory setting. The emamectin benzoate resistant *C. carnea* population demonstrated higher emergence rates in adults, increased fecundity, egg hatchability, and shorter developmental times than the susceptible population. Population growth rates, including intrinsic rate of increase and biotic potential, were also higher in the emamectin benzoate-selected population than in the susceptible population. Another study by Abbas et al. (2014) involved selecting a field-collected population of the common green lacewing *C. carnea* with the insecticide spinosad, and the spinosad-resistant population exhibited a relative fitness advantage, with higher adult emergence rates, fecundity, hatchability, and shorter developmental times as a fitness cost of resistance. The study also found that the spinosad-selected population displayed higher growth rates and biotic potential compared to a susceptible laboratory population. Rodrigo et al. (2019) investigated the susceptibility of different lacewing species, including *Ceraeochrysa cincta*, *Ceraeochrysa cubana*, *Ceraeochrysa paraguaria*, and *Chrysoperla externa* (Neuroptera: Chrysopidae), to bifenthrin, chlorpyrifos, and imidacloprid insecticides in citrus orchards managed under both organic and conventional pest management systems. They found that *C. cincta* populations from conventional management systems had lower egg-hatching rates but faster life cycles, i.e.*,* shorter egg and larval developmental times and higher population growth. Mansoor and Shad (2022a) investigated the resistance development potential of the field-collected *C. carnea*, when subjected to selection with cyromazine and methoxyfenozide, and the results of the study indicate no significant difference between sex ratios and adult mortality in cyromazine and methoxyfenozide selected strains. The studies about cross-resistance patterns and realized heritability also hold significant importance. This exploration spans diverse insecticide classes—like acetamiprid, buprofezin, methoxyfenozide, and nitenpyram—evaluated for their effects on *C. carnea* to understand resistance mechanisms (Mansoor et al., 2017; Mansoor and Shad, 2019; Mansoor and Shad, 2020; Mansoor and Shad, 2022b).

These studies have set the stage for using insecticide-resistant lacewings as a valuable biocontrol agent in controlling important crop pests, especially in cases where chemicals are currently necessary.

## 7 Coleoptera

Despite having over 250,000 species, the Coleoptera order does not include as many agricultural pests as Hemiptera and Lepidoptera. Regardless, pests in this family do enormous damage to agriculture and forestry throughout their life cycles, both as larvae and adults (Patole, 2017). Many of these were pests of storage, wreaking havoc on grain and other seed silos, and also dried plant material. In tropical regions, the maize weevil, *Sitophilus zeamais* Motschulsky (Coleoptera: Curculionidae), is an important pest of stored grains, primarily maize (Ojo and Omoloye, 2016; Nwosu, 2018). Insecticide resistance and fitness studies of this insect have been a major concern all-round year due to the overreliance on pesticides for control (Haddi et al., 2018).

The fitness costs induced by insecticide resistance are a typical assumption in insecticide resistance evolution models. (Vézilier et al., 2013; Cordeiro et al., 2017; Guedes et al., 2017). These costs are most likely the result of an energy imbalance, which diverts energy away from the core physiological processes and towards pesticide resistance (Von Santos et al., 2013; Cordeiro et al., 2017). Despite this, fitness losses associated with pesticide resistance in the absence of insecticides are not ubiquitous, although they often occur (Vézilier et al., 2013). Demographic and competition research using insecticide-susceptible and insecticide-resistant maize weevil strains revealed that certain strains have fitness costs associated with pesticide resistance while others do not (Guedes et al., 2017).

According to Guedes et al. (2009), in both areas, the behavioral patterns of insect movement varied with the population. The different locomotor patterns observed among *S. zeamais* populations may be attributed to the difference in insect metabolism, which can affect insect behavior. However, these differences were not related to the activity of carbohydrate and lipid-metabolizing enzymes in the maize weevil populations studied. Greater body mass and energy storage in *S. zeamais,* resulting in higher respiration rates, were linked to lower fitness costs associated with pesticide resistance (Araújo et al., 2008; Cordeiro et al., 2017; Guedes et al., 2017). Similarly, Araújo et al. (2008) observed that in the absence of insecticides, resistance is typically linked with fitness losses, but prior selection with these chemicals may encourage the evolution of fitness modifier genes that attenuate such costs. In pesticide-resistant strains’ enzymes exhibited higher serine- and cysteine-proteolytic and cellulolytic activity, and kinetic characteristics revealed that cysteine-proteinase and cellulase activities were more essential in reducing the cost of insecticide resistance in maize weevil strains (Araújo et al., 2008).

Colorado potato beetle, *Leptinotarsa decemlineata*, has been the subject of many resistance and fitness studies. *L. decemlineata* strains resistant to OPs and pyrethroids have been discovered in various regions of the world (Freeman et al., 2021). Experiments on lab strains of the beetle with Bt resistance revealed that the resistant lines had a higher fitness cost than their susceptible equivalents. However, the resistance levels decreased dramatically after only five generations without selection pressure (Jisha et al., 2013).

Genetic research and early findings suggest that fitness disadvantages associated with phosphine resistance have been predicted (Mau et al., 2012; Bajracharya, 2013; Collins et al., 2017; Aulicky et al., 2019). In the three species of the red flour beetle (*Tribolium castaneum*), the lesser grain borer (*Rhyzopertha dominica*), and the saw-toothed grain beetle (*Oryzaephilus surinamensis*), resistance to phosphine is associated with a fitness cost, which can potentially compromise the fixation and dispersal of resistant genotypes. In some cases, years of rigorous selection for phosphine resistance may have favored suppressing the negative consequences, reducing the fitness costs normally associated with it. In pyrethroid-resistant populations of *S. zeamais*, such mitigation has already been described (Cordeiro et al., 2017; Guedes et al., 2017). Resistance prevalence data also benchmark against which management performance can be measured (Daglish et al., 2015; Bughio and Wilkins, 2021). The prevailing mechanism of phosphine resistance in these insects involves a reduced uptake of the fumigant, a process designated as active exclusion (Nguyen et al., 2015). This process may be closely related to the insect respiration rate, which is not usually determined in studies of resistance to phosphine. Respiration is also a good index of the physiological responses of insects to the environment to which it is exposed (Wang et al., 2020d). The assumption of an insecticide resistance fitness cost is based on the acquisition of adaptability to a new environment, one contaminated with insecticides. Current findings support previous findings that resistant *S. oryzae* and *S. zeamais* populations had lower fecundity and growth rates than susceptible populations (Daglish et al., 2014). Differences in the biological parameters affecting the growth rate of insect populations are fundamental to insecticide resistance management because, in this case, the frequency of resistant individuals can decrease with time (Gould et al., 2018). However, resistant strains may have a fitness advantage under particular conditions, and resistant individuals may not decrease over time (Guedes et al., 2009). The population of each species produced carbon dioxide, and the instantaneous rate of population expansion (*r*_i_) was associated with their resistance ratios at LC_50_. There was a strong correlation between respiration rate and phosphine resistance in all species of stored-product pests. Populations with lower carbon dioxide generation had a greater resistance ratio (Wang et al., 2020d), indicating that the decreased respiration rate represents the physiological foundation of resistance to phosphine via lowering the insect’s fumigant intake (Nguyen et al., 2015; Alnajim, 2020). In contrast, groups with a greater *r*_i_ exhibited lower resistance ratios, which may imply a reduced reproduction rate in resistant populations compared to susceptible ones. This lends weight to the idea that allocating energy for forming defense mechanisms against pesticides would lower the reproductive potential of resistant populations (Cordeiro et al., 2017). Unlike susceptible populations, resistant populations of *S. oryzae*, *S. zeamais*, and *Cryptolestes ferrugineus* exhibited decreased fertility and growth rates (Daglish et al., 2014; Chakraborty and Madhumathi, 2020). In the absence of insecticides, pesticide resistance is typically linked with an increase in the cost of adaptation. Due to the reallocation of resources from a fundamental physiological function to the defense against insecticides, favoring their survival at the price of their reproduction, these expenditures diminish the reproductive success of resistant individuals (Bass et al., 2014; Cordeiro et al., 2017). Adaptive costs associated with pesticide resistance have been documented in some populations of *S. zeamais* (Cordeiro et al., 2017; Guedes et al., 2017). Consumption of oxygen and generation of carbon dioxide is related to metabolism and might indicate energy expenditures (Chown et al., 2016; Abbas et al., 2020). Modifications in fat body shape reflect the availability and mobilization of energy reserves for the individual’s maintenance, resulting in its survival when exposed to harmful chemicals (Cordeiro et al., 2017). These patterns and study techniques were created in investigations with populations of pyrethroid-resistant maize weevils (Cordeiro et al., 2017), where the resistant population demonstrated greater respiration rate and body mass than the susceptible and another resistant population, demonstrating a reduction of fitness costs. Araújo et al. (2008) report that populations of *S. zeamais* with varying degrees of pesticide sensitivity accumulate and mobilize energy stores differently. These changes enable *S. zeamais* to survive hazardous chemicals better without sacrificing reproductive fitness. Enzymatic tests using carbohydrate- and lipid-metabolizing enzymes measured the activity levels of trehalase, glycogen phosphorylase, lipase, glycosidase, amylase, and respirometry bioassays in two insecticide-resistant populations with (resistance cost) or without (no-cost resistance) associated fitness cost and an insecticide-susceptible population. The group with no cost resistance had much greater body mass and respiration rate than the other two populations, which were comparable. The levels of permethrin resistance, body mass, and respiration rates reported in the above research support those of Guedes et al. (2017), who postulated that an insecticide-resistant population’s greater respiration rate, body mass, and energy reserves reduce the fitness penalty generally associated with pesticide resistance. This mitigation maintains pesticide resistance mechanisms without interfering with other physiological functions, like reproduction.

Amylase cleaves starch and similar polysaccharides, enabling their ultimate storage and utilization as an energy source. It then becomes the substrate of another set of carbohydrases (such as glycosidase and trehalase) that hydrolyze oligosaccharides and disaccharides (Harrison et al., 2012). Trehalase, found in many insects, breaks down trehalose into glucose (Dolezal and Toth, 2014; Kaur et al., 2014; Rodríguez et al., 2015). Lipases are involved in lipid digestion and mobilization, but our *in vitro* bioassays could not tell the difference (Dolezal and Toth, 2014; Jiang et al., 2016). Lipid hydrolysis was more efficient in the resistant population with a cost, whereas starch digestion was more efficient in the resistant group with no cost. The increased activity of amylases in the resistant no-cost population indicates their superior efficiency in extracting energy from food and storing it, resulting in a bigger body mass (Cordeiro et al., 2017). The increased trehalose activity in the resistant cost group likely indicates better energy mobilization, reducing the likelihood of its buildup and resulting rise in body mass. It is difficult to determine the significance of increased lipase activity in the resistant cost population since the enzyme source was the whole insect body, and the lipase classes (involved in fat digestion or lipid mobilization) were not differentiated. Nevertheless, given the increased trehalase activity and the obvious existence of resistance costs in the resistant cost group (Cordeiro et al., 2017; Guedes et al., 2017), the higher lipase activity reported in this population indicates more lipid mobilization. The no-cost-resistant population has a bigger body mass than the cost-resistant population. It is more resistant and likely needs more energy mobilization to maintain its higher pesticide resistance (Cordeiro et al., 2017; Guedes et al., 2017).

## 8 Lepidoptera

The caterpillars and rarely moths of the order Lepidoptera are known for their herbivory on vast crops. These insects feed using chewing and biting mouthparts and devour vegetation through nibbling, boring, mining, etc. Hence, large-scale systemic, as well as contact insecticides, have been used to manage lepidopterans, eventually developing resistance against them (Hafeez et al., 2022; Kenis et al., 2023). Recently, genetically modified crops have also developed resistance in insects despite the rate of resistance development being slower than that of insecticides (Fernandez-Cornejo et al., 2014). For example, Bt cotton, which produces toxins against cotton bollworms upon ingestion, has developed resistance to these insects, which is somewhat counteracted or delayed by their reduced fitness (Carrière et al., 2019). *Pectinophora gossypiella* is resistant to the Cry1Ac toxin generated by Bt cotton due to a gene mutation that inhibits cadherin protein binding to Cry1Ac (Fabrick et al., 2014; Wang et al., 2019c). Furthermore, its resistance to Cry2Ab is due to a mutation of the ATP-binding cassette transporter gene (Heckel, 2015; Mathew et al., 2018). Higher levels of gossypol in traditional cottonseed enhanced the fitness cost of *P. gossypiella* to Bt cotton by affecting survival (Carrière et al., 2019). Also, using biological control agents, multiple toxins, non-Bt refuge and defensive plant compounds, such as gossypol, will reduce/delay the resistance development (Carrière et al., 2019). The insect populations with the non-recessive mode of inheritance of resistance face an early decline in susceptibility, yet high fitness costs associated with them also help delay it (Ullah et al., 2020b).

Research conducted on the widespread fall armyworm, *S. frugiperda*, has documented numerous instances of its resistance and consequent detrimental impact on its biological fitness to a variety of Bt toxins and multiple insecticidal classes, namely, benzoylureas and pyrethroids. Additionally, relatively new insecticides like spinetoram and metaflumizone have shown the same effects on this insect (Barbosa et al., 2020). One example of organophosphate is chlorpyriphos, a widely used insecticide against *S. frugiperda.* Its resistant strains exhibited lower survival, developmental period, pupal weights, fecundity, and fertility life table parameters (Garlet et al., 2021). In another wide-scale used diamide insecticide, chlorantraniliprole, the field-collected resistant strain showed reduced fitness based on population history parameters compared to the near-isogenic resistant strain. This highlighted the importance of using the suitable genetic background of the crop for fitness costs-related studies (Elias et al., 2022). Shan et al. (2021) reported the trade-offs between chlorantraniliprole resistance and chemical communication as well as the fitness cost of insecticide resistance in *P. xylostella*.

Due to its resistance to 97 pesticides, the diamondback moth, *Plutellaxylostella*, is regarded as the most difficult pest of the Brassicaceae family to eradicate (IRAC, 2020). In several nations, it is resistant to almost all key insecticidal groups, including Bt. Since 2010, Japanese farmers have used the rotation of pesticides with varied modes of action for resistance management. Nonetheless, this technique produces variations in their mortalities (Uesugi, 2021) (IRAC 2019). Fitness costs related to using insecticides could be a possible reason for this disparity, resulting in varying susceptibility against the different modes of action affecting resistance stability (Jouzani et al., 2017). Bt, spinosyns, pyridalyl, and aver-mectins/milbemycins may become key components of the rotation approach because their high fitness costs lead to steady and high susceptibility (Jouzani et al., 2017; Yin et al., 2019; Uesugi, 2021; Kumar et al., 2022).

## 9 Diptera

The dipterans represent agricultural, domestic and medically important pests, which have suffered immense insecticidal exposure. *Musca domestica* is a classic example of insecticidal resistance reported in insects (Roca-Acevedo et al., 2023). It is broadly resistant to all the major insecticidal groups, such as organochlorides, organophosphates, carbamates, and pyrethroids, and has been linked to its reduced fitness for one or more traits. Some such examples are spiromesifen (Alam et al., 2020), chlorantraniliprole (Shah and Shad, 2020), spinosad (Khan, 2018), pyriproxyfen (Shah et al., 2015), methoxyfenozide (Shah et al., 2017), fipronil (Abbas et al., 2016a), lambda-cyhalothrin (Abbas et al., 2016b) and imidacloprid (Abbas et al., 2015a). Houseflies develop resistance to insecticides to varying degrees depending on the chemical and its mode of action, which is partly a function of how often and for how long they are exposed to the chemicals. In the absence of insecticides, there will be a reversion of resistance in the process of regaining reduced fitness (Abbas et al., 2015b).

The susceptibility also varies according to season, frequency of application, and the insecticidal group due to changing biological parameters or termination of insecticide exposure in off season (Abbas et al., 2015b). Additionally, pesticide cross-resistance and multiple-resistance contribute to a decrease in sensitivity. Cross-resistance between organophosphates and pyrethroids owing to the presence of detoxifying enzymes such as esterases is one example (Muthusamy et al., 2014). The long use of pyrethroids and DDT to control houseflies has resulted in resistance build-up, mainly attributed to the *kdr* gene family involving multiple alleles (Bass et al., 2014). Interestingly, the lowest resistance imparting *kdr-his* allele is present in two-third of flies’ population in the United States. In contrast, *kdr* and *super-kdr* alleles were less prevalent despite imparting higher resistance (Rinkevich et al., 2013). This may be due to decreased fitness costs associated with *kdr-his* alleles, showing that the biology of flies plays a role in disseminating some resistance-conferring alleles (Sun et al., 2016; Hanai et al., 2018).

There have been reports of resistance to many organophosphate and carbamate chemicals (Low et al., 2013) in *Liriomyza sativae* (Askari-Saryazdi et al., 2015), *Drosophila melanogaster* (Hubhachen et al., 2022), *B. tabaci* (Renault et al., 2023) with a reduction or increase in acetylcholinesterase sensitivity as the major resistance mechanism. Metabolic enzymes as the driving force for altered susceptibility is also prevalent, for example, in *M. domestica* against chlorantraniliprole,- utilizing cytochrome P450 monooxygenase and esterase detoxifying enzymes (Shah and Shad, 2020). Interestingly, there was a varied response of detoxifying enzymes on resistance due to differential insecticidal exposure in different life stages of insects. For example, resistance to chlorpyriphos was more observed in the larval than in the adult stage in *Anopheles gambiae* and *L. sativae* (Askari-Saryazdi et al., 2015). Differential entry routes and chemical properties can force insects to cope with these stress conditions differently in diverse life stages at the cost of their biological fitness (Mastrantonio et al., 2019).

Fruit flies, which are polyphagous, multivoltine, and agriculturally important insect pests, are resistant to various chemicals. One such example is the resistance of a peach fruit fly strain to trichlorfon, an organophosphate, which showed a reduction in relative fitness up to 0.52 (Abubakar et al., 2021). A similar trend was also found for *Bactrocera dorsalis* concerning this specific chemical (Chen et al., 2015). The reduced fitness traits included fecundity, pupal weight, reduced larval duration, number of future larvae, and net reproductive rate, with no difference in biotic potential and intrinsic rate relative to unselected strains (Chen et al., 2015).

It is known that pesticide resistance severely influences mosquito fitness overall and has a number of detrimental impacts on its growth and reproduction features (Diniz et al., 2015; Osoro et al., 2021; Gonzalez-Santillan et al., 2022; Parker-Crockett et al., 2022). The insecticides temephos and deltamethrin are not effective against the laboratory-created strain of *Aedes aegypti* known as *Aedes* Rio, which was developed from field mosquito populations of Rio de Janeiro. Compared to Rockefeller’s laboratory reference strain, pleiotropic effects that led to a fitness cost that metabolic and target site resistance mechanisms may have induced evolved in the original populations. However, due to this diminished fitness, the *A. aegypti* Rio strain did not become infected with or spread the Zika virus (Dos Santos et al., 2020).

The understanding of molecular biology has assisted us in predicting the costs associated with various resistance mechanisms. Bass (2017) critically reviewed the literature on fitness costs in the absence of pesticides. The resistance alleles can develop from pre-existing polymorphisms, and sexual differences can also sustain resistance-associated variation. For resistance induced by *kdr* and *RDL* genes, heterozygous (RS) male *A. gambiae* demonstrated greater mating success than their homozygous resistant (RR) equivalents. This shows that in this instance, homozygous target site resistance has a cost in terms of decreased mating success (Platt et al., 2015).

Most arthropod species have complex connections with symbiotic bacteria and depend on microorganisms for reproduction, development, metabolism, and immunity (Douglas, 2015). The disruption of these commensal bacteria may have detrimental consequences on the physiology of insects, leading to mortality or diminished fitness. For instance, adult tsetse flies given a blood meal containing the antibiotic tetracycline and lice fed on four different medicines exhibited immediate mortality (Weiss et al., 2012). In growing nymphs of the omnivorous American cockroach, removing symbiotic bacteria from the stomach with metronidazole impeded weight gain (Bauer et al., 2015). The elimination of bacterial symbionts may alter not just the longevity and fertility of insects but also their susceptibility to insecticides (Pietri and Liang, 2018). Insecticide resistance in the bean bug, *Riptortus pedestris*, and oriental fruit fly, *B. dorsalis*, has been linked to the synthesis of detoxifying enzymes by Proteobacteria in the midgut, and antibiotic treatments may restore sensitivity to resistant individuals (Kikuchi et al., 2012; Cheng et al., 2017). According to (Pietri et al., 2018), microbiota contributes to both physiological and evolutionary elements of pesticide resistance, suggesting that targeting this community might be a useful tactic. Meanwhile, the gut microbiota also acquired insecticide resistance, contributing to host resistance (Engel and Moran, 2013). Pesticide resistance altered the makeup of the gut microbiota of cockroaches, altering the growth and development of insect hosts. Prior research has examined insecticide resistance mechanisms in German cockroaches regarding target site insensitivity, epidermal permeability, behavioral resistance, and metabolic detoxification (Boopathy et al., 2022), indicating that the gut microbiota may potentially play a part in the resistance process.

Cai et al. (2020) reported that cypermethrin resistance in *B. germanica* affected the development of ovaries and the expression of proteins with different functions in the ovaries, resulting in fecundity defects in the cypermethrin-resistant (R) strain of *B*. *germanica*, which reflected as fitness disadvantages. The R strain of *B. germanica* showed an even greater ootheca shedding rate, a much lower number of hatched and surviving nymphs, a significantly higher percentage of females in the population, and aberrant ovarian development than the sensitive (S) strains. Consequently, the metabolic variations required to overcome the negative effects of pesticides might result in an energy exchange that changes energy allocation and, eventually, the insect’s fundamental requirements. The fitness cost caused by pesticide resistance is crucial for preventing the development of resistance.

This unique integrated pest management (IPM) technique might be used to develop pesticide synergists that inhibit insecticide metabolism in pest microbiota and biocontrol agents (Zhang et al., 2018b; Zhang and Yang, 2019). Given the substantial fitness costs associated with resistance, insecticide rotation may be a viable resistance control strategy. Identifying fitness costs associated with insecticidal resistance may restrict the spread of resistant populations and enable IPM programs to target resistant populations efficiently.

## 10 Fitness advantage

The phenomena of fitness cost or advantage in relation to the resistance of insecticides are familiar in the insect species. Identifying fitness advantages can be useful in deciding integrated pest management by curbing the spread of resistant populations. Fitness costs and resistant individuals spread in the population are directly proportional, thereby becoming an important parameter in decision-making (Kliot and Ghanim, 2012). The major purpose behind investigating the fitness advantage due to insecticide resistance is to monitor the resistance levels over time in environments distinctly exposed to insecticides (Hawkins et al., 2019). Furthermore, if the principal mechanism selected for resistance is known, the genotyping of resistance genes in place and time scales render important assumptions about their fitness advantage (Belinato and Martins, 2016).

The resistant (R) and susceptible (S) *Eriopis connexa* (Germar) populations are crossed, and the performance of the F1 offspring and resistance maintenance over F1, F2, and F3 progenies was assessed (Lira et al., 2016). Compared to the R population, the heterozygous F1 progeny exhibited much higher fecundity and longevity. These findings showed that when beetles from the R population are released and mated with the S population, the field progeny retain the resistance phenotype with an advantage over the parental R population in terms of increased egg production and longer survival. A life table analysis of fenvalerate on the brown planthopper, *N. lugens* indicated that the resistant strain (G4 and G8) demonstrated an improved female ratio, copulation rate, and fecundity. However, the resistant strain had a reduced hatchability. The number of offspring in the G8 generation was higher than that in the G4 generation, and resistant strains in generations G4 and G8 demonstrated a fitness advantage (1.04 and 1.11) (Ling et al., 2011). When an organism faces a niche change and adapts to a new environment, a fitness advantage is a phenomenon that manifests. If the evolutionary pressure lasts for a number of generations, it is possible that other genetic alterations will take place to reduce the negative impacts of the first adaptation and improve it such that there are no longer any fitness costs. The existence of fitness advantages during the laboratory conditions is also important in the evaluation of specific traits.

## 11 Conclusion

Chemical application remains a prevalent strategy for global insect pest control. However, the widespread use of chemical insecticides subjects both intended targets and unintended species to toxic compounds and concurrent stressors. In these challenges, insects must allocate energy and resources for survival and adaptation. Insects counteract the effects of toxic chemicals by employing behavioral, physiological, and genetic defenses. The persistent selective pressure from insecticide usage can lead to resistance development through mechanisms such as upregulation of detoxification genes and target-site mutations.

Numerous studies have underscored the resource-intensive nature of these defense mechanisms, impacting vital biological traits like development and reproduction and other pivotal factors influencing insect fitness. However, future studies should focus on determining the underlying molecular mechanisms of fitness costs associated with insecticide resistance. Such insights can potentially guide the development of effective resistance management strategies for sustainable insect control programs. Furthermore, validating laboratory findings by quantitatively assessing resistance across various fitness matrices within field contexts is imperative. This comprehensive approach is necessary to comprehensively elucidate the intricate interplay between resistance development and fitness costs in agroecosystems at the community level.

## References

[B1] Abbas N. Shad S. A. (2015). Assessment of resistance risk to lambda-cyhalothrin and cross-resistance to four other insecticides in the house fly, *Musca domestica* L.(Diptera: muscidae). Parasitol. Res. 114, 2629–2637. 10.1007/s00436-015-4467-2 25903007

[B2] Abbas N. Mansoor M. M. Shad S. A. Pathan A. K. Waheed A. Ejaz M. (2014). Fitness cost and realized heritability of resistance to spinosad in *Chrysoperla carnea* (neuroptera: chrysopidae). Bull. Entomol. Res. 104 (6), 707–715. 10.1017/S0007485314000522 25033090

[B3] Abbas N. Khan H. Shad S. A. (2015a). Cross-resistance, stability, and fitness cost of resistance to imidacloprid in *Musca domestica* L.,(Diptera: muscidae). Parasitol. Res. 114, 247–255. 10.1007/s00436-014-4186-0 25342464

[B4] Abbas N. Ijaz M. Shad S. A. Khan H. (2015b). Stability of field-selected resistance to conventional and newer chemistry insecticides in the house fly, *Musca domestica* L.(Diptera: muscidae). Neotropical Entomol. 44, 402–409. 10.1007/s13744-015-0290-9 26174963

[B5] Abbas N. Shah R. M. Shad S. A. Azher F. (2016a). Dominant fitness costs of resistance to fipronil in *Musca domestica* linnaeus (Diptera: muscidae). Vet. Parasitol. 226, 78–82. 10.1016/j.vetpar.2016.06.035 27514889

[B6] Abbas N. Shah R. M. Shad S. A. Iqbal N. Razaq M. (2016b). Biological trait analysis and stability of lambda-cyhalothrin resistance in the house fly, *Musca domestica* L.(Diptera: muscidae). Parasitol. Res. 115, 2073–2080. 10.1007/s00436-016-4952-2 26874957

[B7] Abbas W. Withers P. C. Evans T. A. (2020). Water costs of gas exchange by a speckled cockroach and a darkling beetle. Insects 11 (9), 632. 10.3390/insects11090632 32937981 PMC7563770

[B8] Abubakar M. Ali H. Shad S. A. Anees M. Binyameen M. (2021). Trichlorfon resistance: its stability and impacts on biological parameters of *Bactrocera zonata* (Diptera: tephritidae). Appl. Entomol. Zool. 56 (4), 473–482. 10.1007/s13355-021-00754-6

[B9] Alam M. Shah R. M. Shad S. A. Binyameen M. (2020). Fitness cost, realized heritability and stability of resistance to spiromesifen in house fly, *Musca domestica* L.(Diptera: muscidae). Pestic. Biochem. Phys. 168, 104648. 10.1016/j.pestbp.2020.104648 32711758

[B10] Alnajim I. A. (2020). “Comparison of physiological and metabolic changes between phosphine resistant and susceptible strains of Rhyzopertha dominica (Fabricius) and *Tribolium castaneum* (Herbst),” (Perth, Australia: Murdoch University). Doctoral dissertation.

[B11] Amandeep K. Singh N. N. Mukesh K. (2012). Persistent toxicity of insecticides on biotic potential of *Trichogramma brasiliensis* ashmead (Hymenoptera: trichogrammatidae), an egg parasitoid of tomato fruit borer. Indian J. Entomol. 74 (4), 359–365.

[B12] Araújo R. A. Guedes R. N. Oliveira M. G. Ferreira G. H. (2008). Enhanced proteolytic and cellulolytic activity in insecticide-resistant strains of the maize weevil, *Sitophilus zeamais* . J. Stored Prod. Res. 44 (4), 354–359. 10.1016/j.jspr.2008.03.006

[B13] Askari-Saryazdi G. Hejazi M. J. Ferguson J. S. Rashidi M. R. (2015). Selection for chlorpyrifos resistance in *Liriomyza sativae* blanchard: cross-resistance patterns, stability and biochemical mechanisms. Pestic. Biochem. Phys. 124, 86–92. 10.1016/j.pestbp.2015.05.002 26453235

[B14] Aulicky R. Stejskal V. Frydova B. (2019). Field validation of phosphine efficacy on the first recorded resistant strains of *Sitophilus granarius* and *Tribolium castaneum* from the Czech Republic. J. Stored Prod. Res. 81, 107–113. 10.1016/j.jspr.2019.02.003

[B15] Bagheri A. Askari Seyahooei M. Fathipour Y. Famil M. Koohpayma F. Mohammadi-Rad A. (2019). Ecofriendly managing of *Helicoverpa armigera* in tomato field by releasing *Trichogramma evanescence* and *Habrobracon hebetor* . J. Crop Prot. 8, 11–19.

[B16] Bajracharya N. S. (2013). Phosphine resistance in stored-product insect pests: Management and fitness cost. Oklahoma State University.

[B17] Banazeer A. Afzal M. B. Hassan S. Ijaz M. Shad S. A. Serrão J. E. (2021). Status of insecticide resistance in *Plutella xylostella* (linnaeus)(Lepidoptera: plutellidae) from 1997 to 2019: cross-resistance, genetics, biological costs, underlying mechanisms, and implications for management. Phytoparasitica 50, 465–485. 10.1007/s12600-021-00959-z

[B18] Barbosa M. G. Andre T. P. Pontes A. D. Souza S. A. Oliveira N. R. Pastori P. L. (2020). Insecticide rotation and adaptive fitness cost underlying insecticide resistance management for *Spodoptera frugiperda* (Lepidoptera: noctuidae). Neotrop Entomol. 49, 882–892. 10.1007/s13744-020-00800-y 32632568

[B19] Barbosa M. G. Souza S. A. André T. P. Pontes A. D. Teixeira C. S. Pereira F. F. (2021). Do fall armyworm’s Metaflumizone resistante populations affect the activity of *Trichogramma pretiosum*? Braz. J. Biol. 83, e245273. 10.1590/1519-6984.245273 34669790

[B20] Basit M. (2019). Status of insecticide resistance in *Bemisia tabaci*: resistance, cross-resistance, stability of resistance, genetics and fitness costs. Phytoparasitica 47 (2), 207–225. 10.1007/s12600-019-00722-5

[B21] Bass C. Puinean A. M. Zimmer C. T. Denholm I. Field L. M. Foster S. P. (2014). The evolution of insecticide resistance in the peach potato aphid, *Myzus persicae* . Insect Biochem. Mol. Biol. 51, 41–51. 10.1016/j.ibmb.2014.05.003 24855024

[B22] Bass C. (2017). Does resistance really carry a fitness cost? Curr. Opin. Insect Sci. 21, 39–46. 10.1016/j.cois.2017.04.011 28822487 PMC5972224

[B23] Bauer E. Lampert N. Mikaelyan A. Köhler T. Maekawa K. Brune A. (2015). Physicochemical conditions, metabolites and community structure of the bacterial microbiota in the gut of wood-feeding cockroaches (Blaberidae: panesthiinae). FEMS Microbiol. Ecol. 91 (2), 1–14. 10.1093/femsec/fiu028 25764554

[B24] Belinato T. A. Martins A. J. (2016). Insecticide resistance and fitness cost. InTech. 10.1093/femsec/fiu028

[B25] Bielza P. (2016). “Insecticide resistance in natural enemies,” in Advances in insect control and resistance management. Editors Horowitz A. Ishaaya I. (Cham: Springer), 313–329. 10.1007/978-3-319-31800-4-16

[B26] Bielza P. Balanza V. Cifuentes D. Mendoza J. E. (2020). Challenges facing arthropod biological control: identifying traits for genetic improvement of predators in protected crops. Pest Manag. Sci. 76 (11), 3517–3526. 10.1002/ps.5857 32281233

[B27] Biondi A. Chailleux A. Lambion J. Han P. Zappalà L. Desneux N. (2013). Indigenous natural enemies attacking *Tuta absoluta* (Lepidoptera: gelechiidae) in southern France. Egypt J. Biol. Pest Control 23 (1), 117–121.

[B28] Biondi A. Campolo O. Desneux N. Siscaro G. Palmeri V. Zappalà L. (2015). Life stage-dependent susceptibility of *Aphytis melinus* DeBach (Hymenoptera: aphelinidae) to two pesticides commonly used in citrus orchards. Chemosphere 128, 142–147. 10.1016/j.chemosphere.2015.01.034 25698292

[B29] Boopathy B. Rajan A. Radhakrishnan M. (2022). Ozone: an alternative fumigant in controlling the stored product insects and pests: a status report. Ozone Sci. Eng. 44 (1), 79–95. 10.1080/01919512.2021.1933899

[B30] Brenner R. J. Kramer R. D. (2019). “Cockroaches (blattaria),” in Medical and veterinary entomology (United States: Academic Press), 61–77.

[B31] Bughio F. M. Wilkins R. M. (2021). Fitness in a malathion resistant *Tribolium castaneum* strain; feeding, growth and digestion. J. Stored Prod. Res. 92, 101814. 10.1016/j.jspr.2021.101814

[B32] Cai T. Huang Y. H. Zhang F. (2020). Ovarian morphological features and proteome reveal fecundity fitness disadvantages in β-cypermethrin-resistant strains of *Blattella germanica* (L.)(Blattodea: blattellidae). Pestic. Biochem. Phys. 170, 104682. 10.1016/j.pestbp.2020.104682 32980072

[B33] Carrière Y. Yelich A. J. Degain B. A. Harpold V. S. Unnithan G. C. Kim J. H. (2019). Gossypol in cottonseed increases the fitness cost of resistance to Bt cotton in pink bollworm. Crop Prot. 126, 104914. 10.1016/j.cropro.2019.104914

[B34] Castellanos N. L. Haddi K. Carvalho G. A. de Paulo P. D. Hirose E. Guedes R. N. (2019). Imidacloprid resistance in the neotropical brown stink bug *Euschistus heros*: selection and fitness costs. J. Pest Sci. 92, 847–860. 10.1007/s10340-018-1048-z

[B35] Chailleux A. Biondi A. Han P. Tabone E. Desneux N. (2013). Suitability of the pest–plant system *Tuta absoluta* (Lepidoptera: gelechiidae)–tomato for *Trichogramma* (Hymenoptera: trichogrammatidae) parasitoids and insights for biological control. J. Econ. Entomol. 106 (6), 2310–2321. 10.1603/ec13092 24498728

[B36] Chakraborty D. Madhumathi T. (2020). Present scenario of insecticide resistance in Rusty grain beetle, *Cryptolestes ferrugenius* (stephens) to malathion and deltamethrin in Andhra Pradesh, India. Int. J. Curr. Microbiol. App Sci. 9 (6), 4180–4188. 10.20546/ijcmas.2020.906.489

[B37] Chappell T. M. Huseth A. S. Kennedy G. G. (2019). Stability of neonicotinoid sensitivity in *Frankliniella fusca* populations found in agroecosystems of the southeastern USA. Pest Manag. Sci. 75 (6), 1539–1545. 10.1002/ps.5319 30610765

[B38] Chen L. Liu X. Wu S. Zhu Y. Zeng L. Lu Y. (2015). A comparative study of the population biology of trichlorfon-resistant strains of the oriental fruit fly, *Bactrocera dorsalis* (Diptera: Tephritdae). Acta Entomol. Sin. 58 (8), 864–871.

[B39] Cheng J. Lee X. Gao W. Chen Y. Pan W. Tang Y. (2017). Effect of biochar on the bioavailability of difenoconazole and microbial community composition in a pesticide-contaminated soil. Appl. Soil Ecol. 121, 185–192. 10.1016/j.apsoil.2017.10.009

[B40] Cheng S. Lin R. Wang L. Qiu Q. Qu M. Ren X. (2018). Comparative susceptibility of thirteen selected pesticides to three different insect egg parasitoid *Trichogramma* species. Ecotoxicol. Environ. Saf. 166, 86–91. 10.1016/j.ecoenv.2018.09.050 30248565

[B41] Chong H. K. Peng M. H. Liu K. L. Singham G. V. Neoh K. B. (2022). Role of social aggregation in the fitness cost of pyrethroid-resistant German cockroaches. J. Appl. Entomol. 146 (10), 1320–1332. 10.1111/jen.13070

[B42] Chown S. L. Haupt T. M. Sinclair B. J. (2016). Similar metabolic rate-temperature relationships after acclimation at constant and fluctuating temperatures in caterpillars of a sub-Antarctic moth. J. Insect Phys. 85, 10–16. 10.1016/j.jinsphys.2015.11.010 26592773

[B43] Collins P. J. Falk M. G. Nayak M. K. Emery R. N. Holloway J. C. (2017). Monitoring resistance to phosphine in the lesser grain borer, *Rhyzopertha dominica*, in Australia: a national analysis of trends, storage types and geography in relation to resistance detections. J. Stored Prod. Res. 70, 25–36. 10.1016/j.jspr.2016.10.006

[B44] Cordeiro E. M. Corrêa A. S. Rosi‐Denadai C. A. Tomé H. V. Guedes R. N. (2017). Insecticide resistance and size assortative mating in females of the maize weevil (*Sitophilus zeamais*). Pest Manag. Sci. 73 (5), 823–829. 10.1002/ps.4437 27624414

[B45] Daglish G. J. Nayak M. K. Pavic H. (2014). Phosphine resistance in *Sitophilus oryzae* (L.) from eastern Australia: inheritance, fitness and prevalence. J. Stored Prod. Res. 59, 237–244. 10.1016/j.jspr.2014.03.007

[B46] Daglish G. J. Nayak M. K. Pavic H. Smith L. W. (2015). Prevalence and potential fitness cost of weak phosphine resistance in *Tribolium castaneum* (Herbst) in eastern Australia. J. Stored Prod. Res. 61, 54–58. 10.1016/j.jspr.2014.11.005

[B47] Deguine J. P. Aubertot J. N. Flor R. J. Lescourret F. Wyckhuys K. A. Ratnadass A. (2021). Integrated pest management: good intentions, hard realities. A review. Agron. Sustain Dev. 41 (3), 38. 10.1007/s13593-021-00689-w

[B48] Dingha B. Jackai L. Monteverdi R. H. Ibrahim J. (2013). Pest control practices for the German cockroach (blattodea: blattellidae): a survey of rural residents in North Carolina. Fla. Entomol. 96 (3), 1009–1015. 10.1653/024.096.0339

[B49] Diniz D. F. Melo-Santos M. A. Santos E. M. Beserra E. B. Helvecio E. de Carvalho-Leandro D. (2015). Fitness cost in field and laboratory *Aedes aegypti* populations associated with resistance to the insecticide temephos. Parasites vectors 8 (1), 662–665. 10.1186/s13071-015-1276-5 26715037 PMC4696322

[B50] Dolezal A. G. Toth A. L. (2014). Honey bee sociogenomics: a genome-scale perspective on bee social behavior and health. Apidologie 45, 375–395. 10.1007/s13592-013-0251-4

[B51] Dos Santos C. R. de Melo Rodovalho C. Jablonka W. Martins A. J. Lima J. B. dos Santos Dias L. (2020). Insecticide resistance, fitness and susceptibility to Zika infection of an interbred *Aedes aegypti* population from Rio de Janeiro, Brazil. Parasites Vectors 13 (1), 293–294. 10.1186/s13071-020-04166-3 32513248 PMC7281914

[B52] Douglas A. E. (2015). Multiorganismal insects: diversity and function of resident microorganisms. Annu. Rev. Entomol. 60, 17–34. 10.1146/annurev-ento-010814-020822 25341109 PMC4465791

[B53] Du W. M. Xu J. Hou Y. Y. Lin Y. Zang L. S. Yang X. (2018). *Trichogramma* parasitoids can distinguish between fertilized and unfertilized host eggs. J. Pest Sci. 91, 771–780. 10.1007/s10340-017-0919-z

[B54] Eggleston P. A. (2017). Cockroach allergy and urban asthma. J. Allergy Clin. Immunol. Pract. 140 (2), 389–390. 10.1016/j.jaci.2017.04.033 28528788

[B55] El-Arnaouty S. A. Galal H. H. Afifi A. I. Beyssat V. Pizzol J. Desneux N. (2014). Assessment of two *Trichogramma* species for the control of *Tuta absoluta* in North African tomato greenhouses. Afr. Entomol. 22 (4), 801–809. 10.4001/003.022.0410

[B56] Elias O. P. F. Hideo K. R. Omoto C. Sartori G. A. (2022). Fitness costs associated with chlorantraniliprole resistance in *Spodoptera frugiperda* (Lepidoptera: noctuidae) strains with different genetic backgrounds. Pest Manag. Sci. 78 (3), 1279–1286. 10.1002/ps.6746 34854222

[B57] Engel P. Moran N. A. (2013). The gut microbiota of insects–diversity in structure and function. FEMS Microbiol. Rev. 37 (5), 699–735. 10.1111/1574-6976.12025 23692388

[B58] Fabrick J. A. Ponnuraj J. Singh A. Tanwar R. K. Unnithan G. C. Yelich A. J. (2014). Alternative splicing and highly variable cadherin transcripts associated with field-evolved resistance of pink bollworm to Bt cotton in India. PloS One 9 (5), e97900. 10.1371/journal.pone.0097900 24840729 PMC4026531

[B59] Fernandez-Cornejo J. Wechsler S. Livingston M. Mitchell L. (2014). Genetically engineered crops in the United States. United States: USDA-ERS Economic Research Report.

[B60] Freeman J. C. Smith L. B. Silva J. J. Fan Y. Sun H. Scott J. G. (2021). Fitness studies of insecticide resistant strains: lessons learned and future directions. Pest Manag. Sci. 77 (9), 3847–3856. 10.1002/ps.6306 33506993

[B61] Fu B. Li Q. Qiu H. Tang L. Zeng D. Liu K. (2018). Resistance development, stability, cross-resistance potential, biological fitness and biochemical mechanisms of spinetoram resistance in *Thrips hawaiiensis* (thysanoptera: thripidae). Pest Manage Sci. 74 (7), 1564–1574. 10.1002/ps.4887 29427375

[B62] Fu B. Tao M. Xue H. Jin H. Liu K. Qiu H. (2022). Spinetoram resistance drives interspecific competition between *Megalurothrips usitatus* and *Frankliniella intonsa* . Pest Manag. Sci. 78 (6), 2129–2140. 10.1002/ps.6839 35170208

[B63] Gao C. F. Ma S. Z. Shan C. H. Wu S. F. (2014). Thiamethoxam resistance selected in the western flower thrips *Frankliniella occidentalis* (thysanoptera: thripidae): cross-resistance patterns, possible biochemical mechanisms and fitness costs analysis. Pestic. Biochem. Phys. 114, 90–96. 10.1016/j.pestbp.2014.06.009 25175655

[B64] Garlet C. G. Moreira R. P. Gubiani P. D. Palharini R. B. Farias J. R. Bernardi O. (2021). Fitness cost of chlorpyrifos resistance in *Spodoptera frugiperda* (Lepidoptera: noctuidae) on different host plants. Environ. Entomol. 50 (4), 898–908. 10.1093/ee/nvab046 34018549

[B65] George J. Glover J. P. Gore J. Crow W. D. Reddy G. V. (2021). Biology, ecology, and pest management of the tarnished plant bug, *Lygus lineolaris* (Palisot de Beauvois) in southern row crops. Insects 12 (9), 807. 10.3390/insects12090807 34564247 PMC8465932

[B66] Gontijo L. Cascone P. Giorgini M. Michelozzi M. Rodrigues H. S. Spiezia G. (2019). Relative importance of host and plant semiochemicals in the foraging behavior of *Trichogramma achaeae*, an egg parasitoid of *Tuta absoluta* . J. Pest Sci. 92 (4), 1479–1488. 10.1007/s10340-019-01091-y

[B67] Gonzalez-Santillan F. J. Contreras-Perera Y. Davila-Barboza J. A. Juache-Villagrana A. E. Gutierrez-Rodriguez S. M. Ponce-Garcia G. (2022). Fitness cost of sequential selection with deltamethrin in *Aedes aegypti* (Diptera: culicidae). J. Med. Entomol. 59 (3), 930–939. 10.1093/jme/tjac032 35389486

[B68] Gould F. Brown Z. S. Kuzma J. (2018). Wicked evolution: can we address the sociobiological dilemma of pesticide resistance? Science 360 (6390), 728–732. 10.1126/science.aar3780 29773742

[B69] Grigoraki L. Pipini D. Labbe P. Chaskopoulou A. Weill M. Vontas J. (2017). Carboxylesterase gene amplifications associated with insecticide resistance in *Aedes albopictus*: geographical distribution and evolutionary origin. PLoS Negl. Trop. Dis. 11 (4), e0005533. 10.1371/journal.pntd.0005533 28394886 PMC5398709

[B70] Guedes N. M. Guedes R. N. Ferreira G. H. Silva L. B. (2009). Flight take-off and walking behavior of insecticide-susceptible and–resistant strains of *Sitophilus zeamais* exposed to deltamethrin. Bull. Entomol. Res. 99 (4), 393–400. 10.1017/S0007485309006610 19302721

[B71] Guedes N. M. Guedes R. N. Campbell J. F. Throne J. E. (2017). Mating behaviour and reproductive output in insecticide-resistant and-susceptible strains of the maize weevil (*Sitophilus zeamais*). Ann. App Biol. 170 (3), 415–424. 10.1111/aab.12346

[B72] Guo X. Di N. Chen X. Zhu Z. Zhang F. Tang B. (2019). Performance of *Trichogramma pintoi* when parasitizing eggs of the oriental fruit moth *Grapholita molesta* . Entomol. Gen. 39, 239–249. 10.1127/entomologia/2019/0853

[B73] Gurr G. M. Wratten S. D. Landis D. A. You M. (2017). Habitat management to suppress pest populations: progress and prospects. Annu. Rev. Entomol. 62, 91–109. 10.1146/annurev-ento-031616-035050 27813664

[B74] Haddi K. Valbon W. R. Viteri Jumbo L. O. de Oliveira L. O. Guedes R. N. Oliveira E. E. (2018). Diversity and convergence of mechanisms involved in pyrethroid resistance in the stored grain weevils, *Sitophilus* spp. Sci. Rep. 8 (1), 16361. 10.1038/s41598-018-34513-5 30397209 PMC6218525

[B75] Hafeez M. Li X. Ullah F. Zhang Z. Zhang J. Huang J. (2022). Characterization of indoxacarb resistance in the fall armyworm: selection, inheritance, cross-resistance, possible biochemical mechanisms, and fitness costs. Biology 11 (12), 1718. 10.3390/biology11121718 36552228 PMC9774702

[B76] Hanai D. Hardstone Yoshimizu M. Scott J. G. (2018). The insecticide resistance allele kdr-his has a fitness cost in the absence of insecticide exposure. J. Econ. Entomol. 111 (6), 2992–2995. 10.1093/jee/toy300 30277509

[B77] Hardstone M. C. Huang X. Harrington L. C. Scott J. G. (2014). Differences in development, glycogen, and lipid content associated with cytochrome P450-mediated permethrin resistance in *Culex pipiens quinquefasciatus* (Diptera: culicidae). J. Med. Entomol. 47 (2), 188–198. 10.1603/me09131 20380299

[B78] Harrison J. F. Woods H. A. Roberts S. P. (2012). Ecological and environmental physiology of insects. Oxford: OUP Oxford.

[B79] Hawkins N. J. Bass C. Dixon A. Neve P. (2019). The evolutionary origins of pesticide resistance. Biol. Rev. 94 (1), 135–155. 10.1111/brv.12440 29971903 PMC6378405

[B80] Heckel D. G. (2015). Roles of ABC proteins in the mechanism and management of Bt resistance. InBt resistance: Characterization and strategies for GM crops producing *Bacillus thuringiensis* toxins. Wallingford UK: CABI, 98–106.

[B81] Hou Y. Y. Yang X. Zang L. S. Zhang C. Monticelli L. S. Desneux N. (2018). Effect of oriental armyworm Mythimna separata egg age on the parasitism and host suitability for five *Trichogramma* species. J. Pest Sci. 91, 1181–1189. 10.1007/s10340-018-0980-2

[B82] Hua D. Li X. Yuan J. Tao M. Zhang K. Zheng X. (2023). Fitness cost of spinosad resistance related to vitellogenin in *Frankliniella occidentalis* (Pergande). Pest Manag. Sci. 79 (2), 771–780. 10.1002/ps.7253 36264641

[B83] Huang N. X. Jaworski C. C. Desneux N. Zhang F. Yang P. Y. Wang S. (2020). Long-term and large-scale releases of *Trichogramma* promote pesticide decrease in maize in northeastern China. Entomol. Gen. 40 (4), 331–335. 10.1127/entomologia/2020/0994

[B84] Hubhachen Z. Pointon H. Perkins J. A. Van Timmeren S. Pittendrigh B. Isaacs R. (2022). Resistance to multiple insecticide classes in the vinegar fly *Drosophila melanogaster* (Diptera: drosophilidae) in Michigan vineyards. J. Econ. Entomol. 115 (6), 2020–2028. 10.1093/jee/toac155 36255035

[B85] Jackson G. E. Malloch G. McNamara L. Little D. (2020). Grain aphids (*Sitobion avenae*) with knockdown resistance (kdr) to insecticide exhibit fitness trade-offs, including increased vulnerability to the natural enemy *Aphidius ervi* . Plos One 15 (11), e0230541. 10.1371/journal.pone.0230541 33170844 PMC7654777

[B86] Jactel H. Verheggen F. Thiéry D. Escobar-Gutiérrez A. J. Gachet E. Desneux N. Neonicotinoids Working Group (2019). Alternatives to neonicotinoids. Environ. Int. 129, 423–429. 10.1016/j.envint.2019.04.045 31152983

[B87] Jensen K. Ko A. E. Schal C. Silverman J. (2016). Insecticide resistance and nutrition interactively shape life-history parameters in German cockroaches. Sci. Rep. 6 (1), 28731. 10.1038/srep28731 27345220 PMC4922014

[B88] Jiang Y. P. Li L. Liu Z. Y. You L. L. Wu Y. Xu B. (2016). Adipose triglyceride lipase (Atgl) mediates the antibiotic jinggangmycin-stimulated reproduction in the brown planthopper, *Nilaparvata lugens* Stål. Sci. Rep. 6 (1), 18984. 10.1038/srep18984 26739506 PMC4704046

[B89] Jin R. Mao K. Xu P. Wang Y. Liao X. Wan H. (2021). Inheritance mode and fitness costs of clothianidin resistance in brown planthopper, *Nilaparvata lugens* (Stål). Crop Prot. 140, 105414. 10.1016/j.cropro.2020.105414

[B90] Jisha V. N. Smitha R. B. Benjamin S. (2013). An overview on the crystal toxins from *Bacillus thuringiensis* . Adv. Microbiol. 3 (05), 462–472. 10.4236/aim.2013.35062

[B91] Jouzani G. S. Valijanian E. Sharafi R. (2017). Bacillus thuringiensis: a successful insecticide with new environmental features and tidings. Appl. Microbiol. Biotechnol. 101, 2691–2711. 10.1007/s00253-017-8175-y 28235989

[B92] Kaur R. Kaur N. Gupta A. K. (2014). Structural features, substrate specificity, kinetic properties of insect α-amylase and specificity of plant α-amylase inhibitors. Pestic. Biochem. Phys. 116, 83–93. 10.1016/j.pestbp.2014.09.005 25454524

[B93] Kenis M. Benelli G. Biondi A. Calatayud P. A. Day R. Desneux N. (2023). Invasiveness, biology, ecology, and management of the fall armyworm, *Spodoptera frugiperda* . Entomol. Gen. 43, 187–241. 10.1127/entomologia/2022/1659

[B94] Khan M. A. Ruberson J. R. (2017). Lethal effects of selected novel pesticides on immature stages of *Trichogramma pretiosum* (Hymenoptera: trichogrammatidae). Pest Manag. Sci. 73 (12), 2465–2472. 10.1002/ps.4639 28600808

[B95] Khan H. A. (2018). Spinosad resistance affects biological parameters of *Musca domestica* Linnaeus. Sci. Rep. 8 (1), 14031. 10.1038/s41598-018-32445-8 30232466 PMC6145934

[B96] Kikuchi Y. Hayatsu M. Hosokawa T. Nagayama A. Tago K. Fukatsu T. (2012). Symbiont-mediated insecticide resistance. Proc. Natl. Acad. Sci. 109 (22), 8618–8622. 10.1073/pnas.1200231109 22529384 PMC3365206

[B97] Kliot A. Ghanim M. (2012). Fitness costs associated with insecticide resistance. Pest Manag. Sci. 68 (11), 1431–1437. 10.1002/ps.3395 22945853

[B98] Koo H. N. An J. J. Park S. E. Kim J. I. Kim G. H. (2014). Regional susceptibilities to 12 insecticides of melon and cotton aphid, *Aphis gossypii* (Hemiptera: aphididae) and a point mutation associated with imidacloprid resistance. Crop Prot. 55, 91–97. 10.1016/j.cropro.2013.09.010

[B99] Kumar R. M. Gadratagi B. G. Paramesh V. Kumar P. Madivalar Y. Narayanappa N. (2022). Sustainable management of invasive fall armyworm, *Spodoptera frugiperda* . Agronomy 12 (9), 2150. 10.3390/agronomy12092150

[B100] Lee C. Y. Wang C. Rust M. K. (2021). German cockroach infestations in the world and their social and economic impacts. Biology and management of the German cockroach. Clayton South, Victoria, Australia: CSIRO Publishing, 1–16.

[B101] Lee S. H. Choe D. H. Rust M. K. Lee C. Y. (2022). Reduced susceptibility towards commercial bait insecticides in field German cockroach (blattodea: ectobiidae) populations from California. J. Econ. Entomol. 115 (1), 259–265. 10.1093/jee/toab244 34922391

[B102] Li Z. Feng X. Liu S. S. You M. Furlong M. J. (2016). Biology, ecology, and management of the diamondback moth in China. Annu. Rev. Entomol. 61, 277–296. 10.1146/annurev-ento-010715-023622 26667272

[B103] Li X. Wan Y. Yuan G. Hussain S. Xu B. Xie W. (2017). Fitness trade-off associated with spinosad resistance in *Frankliniella occidentalis* (thysanoptera: thripidae). J. Econ. Entomol. 110 (4), 1755–1763. 10.1093/jee/tox122 28444324

[B104] Li R. Zhu B. Liang P. Gao X. (2022a). Identification of carboxylesterase genes contributing to multi-insecticide resistance in *Plutella xylostella* (L.). Entomol. Gen. 42 (6), 967–976. 10.1127/entomologia/2022/1572

[B105] Li D. Zhi J. Yue W. Zhang T. Liu L. (2022b). Resistance to spinetoram affects host adaptability of *Frankliniella occidentalis* (thysanoptera: thripidae) based on detoxifying enzyme activities and an age-stage-two-sex life table. Environ. Entomol. 51 (4), 780–789. 10.1093/ee/nvac053 35834261

[B106] Liao X. Mao K. Ali E. Jin R. Li Z. Li W. (2019). Inheritance and fitness costs of sulfoxaflor resistance in *Nilaparvata lugens* (Stål). Pest Manag. Sci. 75 (11), 2981–2988. 10.1002/ps.5412 30884104

[B107] Liao X. Xu P. F. Gong P. P. Wan H. Li J. H. (2021). Current susceptibilities of brown planthopper *Nilaparvata lugens* to triflumezopyrim and other frequently used insecticides in China. Insect Sci. 28 (1), 115–126. 10.1111/1744-7917.12764 32043703

[B108] Lin J. Yu X. Q. Wang Q. Tao X. Li J. Zhang S. (2020). Immune responses to *Bacillus thuringiensis* in the midgut of the diamondback moth, *Plutella xylostella* . Dev. Comp. Immunol. 107, 103661. 10.1016/j.dci.2020.103661 32097696

[B109] Ling S. Zhang H. Zhang R. (2011). Effect of fenvalerate on the reproduction and fitness costs of the brown planthopper, Nilaparvata lugens and its resistance mechanism. Pestic. Biochem. Phys. 101 (3), 148–153. 10.1016/j.pestbp.2011.08.009

[B110] Lira R. A. Rodrigues A. R. Torres J. B. (2016). Fitness advantage in heterozygous ladybird beetle *Eriopis connexa* (Germar) resistant to lambda-cyhalothrin. Neotrop Entomol. 45, 573–579. 10.1007/s13744-016-0407-9 27255766

[B111] Lommen S. T. de Jong P. W. Pannebakker B. A. (2017). It is time to bridge the gap between exploring and exploiting: prospects for utilizing intraspecific genetic variation to optimize arthropods for augmentative pest control–a review. Entomol. Exp. Appl. 162 (2), 108–123. 10.1111/eea.12510

[B112] Low V. L. Chen C. D. Lee H. L. Tan T. K. Chen C. F. Leong C. S. (2013). Enzymatic characterization of insecticide resistance mechanisms in field populations of Malaysian *Culex quinquefasciatus* say (Diptera: culicidae). PloS One 8 (11), e79928. 10.1371/journal.pone.0079928 24278220 PMC3836847

[B113] Lu Y. Wu K. Jiang Y. Guo Y. Desneux N. (2012). Widespread adoption of Bt cotton and insecticide decrease promotes biocontrol services. Nature 487 (7407), 362–365. 10.1038/nature11153 22722864

[B114] Ma K. Tang Q. Zhang B. Liang P. Wang B. Gao X. (2019). Overexpression of multiple cytochrome P450 genes associated with sulfoxaflor resistance in *Aphis gossypii* Glover. Pestic. Biochem. Phys. 157, 204–210. 10.1016/j.pestbp.2019.03.021 31153470

[B115] Mahmoodi L. Mehrkhou F. Guz N. Forouzan M. Atlihan R. (2020). Sublethal effects of three insecticides on fitness parameters and population projection of *Brevicoryne brassicae* (Hemiptera: aphididae). J. Econ. Entomol. 113 (6), 2713–2722. 10.1093/jee/toaa193 32918545

[B116] Malathi V. M. Jalali S. K. Gowda D. K. Mohan M. Venkatesan T. (2017). Establishing the role of detoxifying enzymes in field‐evolved resistance to various insecticides in the brown planthopper (*Nilaparvata lugens*) in South India. Insect Sci. 24 (1), 35–46. 10.1111/1744-7917.12254 26200805

[B117] Mansoor M. M. Shad S. A. (2019). Resistance of green lacewing, *Chrysoperla carnea* (stephens), to buprofezin: cross resistance patterns, preliminary mechanism and realized heritability. Biol. Control 129, 123–127. 10.1016/j.biocontrol.2018.10.008

[B118] Mansoor M. M. Shad S. A. (2020). Genetics, cross-resistance and realized heritability of resistance to acetamiprid in generalist predator, *Chrysoperla carnea* (Steph.)(Neuroptera: chrysopidae). Egypt. J. Biol. Pest Control 30 (1), 23. 10.1186/s41938-020-0213-x

[B119] Mansoor M. M. Shad S. A. (2022a). Risk assessment of cyromazine and methoxyfenozide resistance suggests higher additive genetic but lower environmental variation supporting quick resistance development in non-target *Chrysoperla carnea* (Stephens). Environ. Monit. Assess. 194, 66–68. 10.1007/s10661-021-09735-2 34993647

[B120] Mansoor M. M. Shad S. A. (2022b). Methoxyfenozide tolerance in *Chrysoperla carnea*: inheritance, dominance and preliminary detoxification mechanisms. Plos One 17 (3), e0265304. 10.1371/journal.pone.0265304 35316289 PMC8939785

[B121] Mansoor M. M. Abbas N. Shad S. A. Pathan A. K. Razaq M. (2013). Increased fitness and realized heritability in emamectin benzoate-resistant *Chrysoperla carnea* (neuroptera: chrysopidae). Ecotoxicol 22, 1232–1240. 10.1007/s10646-013-1111-8 23975538

[B122] Mansoor M. M. Raza A. B. Abbas N. Aqueel M. A. Afzal M. (2017). Resistance of green lacewing, *Chrysoperla carnea* stephens to nitenpyram: cross-resistance patterns, mechanism, stability, and realized heritability. Pestic. Biochem. Phys. 135, 59–63. 10.1016/j.pestbp.2016.06.004 28043332

[B123] Marchioro C. A. Krechemer F. S. Foerster L. A. (2015). Assessing the total mortality caused by two species of *Trichogramma* on its natural host *Plutella xylostella* (L.) at different temperatures. Neotrop Entomol. 44, 270–277. 10.1007/s13744-014-0263-4 26013271

[B124] Martinez A. J. Ritter S. G. Doremus M. R. Russell J. A. Oliver K. M. (2014). Aphid-encoded variability in susceptibility to a parasitoid. BMC Evol. Biol. 14 (1), 127–210. 10.1186/1471-2148-14-127 24916045 PMC4057601

[B125] Mastrantonio V. Ferrari M. Negri A. Sturmo T. Favia G. Porretta D. (2019). Insecticide exposure triggers a modulated expression of ABC transporter genes in larvae of *Anopheles gambiae* ss. Insects 10 (3), 66. 10.3390/insects10030066 30841542 PMC6468849

[B126] Mathew L. G. Ponnuraj J. Mallappa B. Chowdary L. R. Zhang J. Tay W. T. (2018). ABC transporter mis-splicing associated with resistance to Bt toxin Cry2Ab in laboratory-and field-selected pink bollworm. Sci. Rep. 8 (1), 13531. 10.1038/s41598-018-31840-5 30202031 PMC6131251

[B127] Mau Y. S. Collins P. J. Daglish G. J. Nayak M. K. Ebert P. R. (2012). The rph2 gene is responsible for high level resistance to phosphine in independent field strains of *Rhyzopertha dominica* . Plos One 7 (3), e34027. 10.1371/journal.pone.0034027 22461899 PMC3312893

[B128] Mengoni S. L. Alzogaray R. A. (2018). Deltamethrin-resistant German cockroaches are less sensitive to the insect repellents DEET and IR3535 than non-resistant individuals. J. Econ. Entomol. 111 (2), 836–843. 10.1093/jee/toy009 29415176

[B129] Muthusamy R. Ramkumar G. Karthi S. Shivakumar M. S. (2014). Biochemical mechanisms of insecticide resistance in field population of dengue vector *Aedes aegypti* (Diptera: culicidae). Int. J. Mosq. Res. 1 (2), 1–4.

[B130] Nakao S. Chikamori C. Asuka H. O. Satoshi T. O. (2014). Relationship between pyrethroid Resistance and attacking persimmon in the onion thrips, *Thrips tabaci* (thysanoptera: thripidae). Jpn. J. Appl. Entomol. Zool. 58 (3), 255–262. 10.1303/jjaez.2014.255

[B131] Ndakidemi B. Mtei K. Ndakidemi P. A. (2016). Impacts of synthetic and botanical pesticides on beneficial insects. Agric. Sci. 7 (06), 364–372. 10.4236/as.2016.76038

[B132] Nguyen T. T. Collins P. J. Ebert P. R. (2015). Inheritance and characterization of strong resistance to phosphine in *Sitophilus oryzae* (L.). PloS One 10 (4), e0124335. 10.1371/journal.pone.0124335 25886629 PMC4401577

[B133] Nwosu L. C. (2018). Impact of age on the biological activities of *Sitophilus zeamais* (Coleoptera: curculionidae) adults on stored maize: implications for food security and pest management. J. Econ. Entomol. 111 (5), 2454–2460. 10.1093/jee/toy187 29982773

[B134] Ojo J. A. Omoloye A. A. (2016). Development and life history of *Sitophilus zeamais* (Coleoptera: curculionidae) on cereal crops. Adv. Agric. 2016, 1–8. 10.1155/2016/7836379

[B135] Onstad D. W. Flexner J. L. (2023). “Insect resistance, natural enemies, and density-dependent processes,” in Insect resistance management (United States: Academic Press), 381–399.

[B136] Osoro J. K. Machani M. G. Ochomo E. Wanjala C. Omukunda E. Munga S. (2021). Insecticide resistance exerts significant fitness costs in immature stages of *Anopheles gambiae* in western Kenya. Malar. J. 20 (1), 259. 10.1186/s12936-021-03798-9 34107949 PMC8188659

[B137] Overton K. Hoffmann A. A. Reynolds O. L. Umina P. A. (2021). Toxicity of insecticides and miticides to natural enemies in Australian grains: A review. Insects 12 (2), 187. 10.3390/insects12020187 33671702 PMC7927080

[B138] Park Y. Kim S. Lee S. H. Lee J. H. (2021). Insecticide resistance trait may contribute to genetic cluster change in *Bemisia tabaci* MED (Hemiptera: aleyrodidae) as a potential driving force. Pest Manag. Sci. 77 (7), 3581–3587. 10.1002/ps.6412 33843146

[B139] Parker-Crockett C. Lloyd A. Ramirez D. Connelly C. R. (2022). Impacts of differential mosquito control treatment regimens on insecticide susceptibility status of *Aedes aegypti* (Diptera: culicidae). SN Appl. Sci. 4 (9), 249. 10.1007/s42452-022-05130-9

[B140] Patole S. S. (2017). Review on beetles (Coleopteran): an agricultural major crop pests of the world. Int. J. Life Sci. Sci. Res. 3 (6), 1424–1432. 10.21276/ijlssr.2017.3.6.1

[B141] Pietri J. E. Liang D. (2018). The links between insect symbionts and insecticide resistance: causal relationships and physiological tradeoffs. Ann. Entomol. Soc. Am. 111 (3), 92–97. 10.1093/aesa/say009

[B142] Pietri J. E. Tiffany C. Liang D. (2018). Disruption of the microbiota affects physiological and evolutionary aspects of insecticide resistance in the German cockroach, an important urban pest. PloS One 13 (12), e0207985. 10.1371/journal.pone.0207985 30540788 PMC6291076

[B143] Pires D. Lozano R. E. Menger J. P. Andow D. A. Koch R. L. (2021). Identification of point mutations related to pyrethroid resistance in voltage-gated sodium channel genes in *Aphis glycines* . Entomol. Gen. 41 (3), 243–255. 10.1127/entomologia/2021/1226

[B144] Platt N. Kwiatkowska R. M. Irving H. Diabaté A. Dabire R. Wondji C. S. (2015). Target-site resistance mutations (kdr and RDL), but not metabolic resistance, negatively impact male mating competiveness in the malaria vector *Anopheles gambiae* . Heredity 115 (3), 243–252. 10.1038/hdy.2015.33 25899013 PMC4519523

[B145] Qin Y. Xu P. Jin R. Li Z. Ma K. Wan H. (2021). Resistance of *Nilaparvata lugens* (Hemiptera: delphacidae) to triflumezopyrim: inheritance and fitness costs. Pest Manag. Sci. 77 (12), 5566–5575. 10.1002/ps.6598 34390298

[B146] Qu Y. Chen X. Monticelli L. S. Zhang F. Desneux N. Huijie D. (2020). Parasitism performance of the parasitoid *Trichogramma dendrolimi* on the plum fruit moth *Grapholitha funebrana* . Entomol. Gen. 40 (4), 385–395. 10.1007/s00294-019-01030-5

[B147] Renault D. Elfiky A. Mohamed A. (2023). Predicting the insecticide-driven mutations in a crop pest insect: evidence for multiple polymorphisms of acetylcholinesterase gene with potential relevance for resistance to chemicals. Environ. Sci. Pollut. Res. 30 (7), 18937–18955. 10.1007/s11356-022-23309-w 36219281

[B148] Rinkevich F. D. Leichter C. A. Lazo T. A. Hardstone M. C. Scott J. G. (2013). Variable fitness costs for pyrethroid resistance alleles in the house fly, *Musca domestica*, in the absence of insecticide pressure. Pestic. Biochem. Phys. 105 (3), 161–168. 10.1016/j.pestbp.2013.01.006

[B149] Roca-Acevedo G. Boscaro I. Toloza A. C. (2023). Global pattern of kdr-type alleles in *Musca domestica* (L.). Curr. Trop. Med. Rep. 10 (1), 1–10. 10.1007/s40475-022-00281-6 36569791 PMC9760529

[B150] Rodrigo R. G. Cuervo R. J. B. Anzolut S. P. Takao Y. P. (2019). Pest management systems and insecticide tolerance of lacewings (neuroptera: chrysopidae). J. Econ. Entomol. 112 (3), 1183–1189. 10.1093/jee/toz024 30768668

[B151] Rodríguez E. Weber J. M. Pagé B. Roubik D. W. Suarez R. K. Darveau C. A. (2015). Setting the pace of life: membrane composition of flight muscle varies with metabolic rate of hovering orchid bees. Proc. R. Soc. B Biol. Sci. 282 (1802), 20142232. 10.1098/rspb.2014.2232 PMC4344142 25652831

[B152] Roy D. Bhattacharjee T. Biswas A. Ghosh A. Sarkar S. Mondal D. (2019). Resistance monitoring for conventional and new chemistry insecticides on *Bemisia tabaci* genetic group Asia-I in major vegetable crops from India. Phytoparasitica 47, 55–66. 10.1007/s12600-018-00707-w

[B153] Saeed R. Abbas N. Hafez A. M. (2021). Biological fitness costs in emamectin benzoate-resistant strains of *Dysdercus koenigii* . Entomol. Gen. 41 (3), 267–278. 10.1127/entomologia/2021/1184

[B154] Salas Gervassio N. G. Aquino D. Vallina C. Biondi A. Luna M. G. (2019). A re-examination of *Tuta absoluta* parasitoids in South America for optimized biological control. J. Pest Sci. 92 (4), 1343–1357. 10.1007/s10340-018-01078-1

[B155] Sanada-Morimura S. Fujii T. Chien H. V. Cuong L. Q. Estoy G. F. Jr Matsumura M. (2019). Selection for imidacloprid resistance and mode of inheritance in the brown planthopper, *Nilaparvata lugens* . Pest Manag. Sci. 75 (8), 2271–2277. 10.1002/ps.5364 30701654

[B156] Schmidt-Jeffris R. A. Beers E. H. Sater C. (2021). Meta-analysis and review of pesticide non-target effects on phytoseiids, key biological control agents. Pest Manag. Sci. 77 (11), 4848–4862. 10.1002/ps.6531 34169634

[B157] Shah R. M. Shad S. A. (2020). House fly resistance to chlorantraniliprole: cross resistance patterns, stability and associated fitness costs. Pest Manag. Sci. 76 (5), 1866–1873. 10.1002/ps.5716 31840405

[B158] Shah R. M. Shad S. A. Abbas N. (2015). Mechanism, stability and fitness cost of resistance to pyriproxyfen in the house fly, *Musca domestica* L.(Diptera: muscidae). Pestic. Biochem. Phys. 119, 67–73. 10.1016/j.pestbp.2015.02.003 25868819

[B159] Shah R. M. Shad S. A. Abbas N. (2017). Methoxyfenozide resistance of the housefly, *Musca domestica* L.(Diptera: muscidae): cross-resistance patterns, stability and associated fitness costs. Pest Manag. Sci. 73 (1), 254–261. 10.1002/ps.4296 27098995

[B160] Shan J. Zhu B. Gu S. Liang P. Gao X. (2021). Development of resistance to chlorantraniliprole represses sex pheromone responses in male *Plutella xylostella* (L.). Entomol. Gen. 41 (6), 615–625. 10.1127/entomologia/2021/1359

[B161] Shi R. Zhao H. Tang S. (2014). Global dynamic analysis of a vector-borne plant disease model. Adv. Differ. Equ. 1, 59–16. 10.1186/1687-1847-2014-59

[B162] Sifakis S. Androutsopoulos V. P. Tsatsakis A. M. Spandidos D. A. (2017). Human exposure to endocrine disrupting chemicals: effects on the male and female reproductive systems. Environ. Toxicol. Pharmacol. 51, 56–70. 10.1016/j.etap.2017.02.024 28292651

[B163] Singarayan V. T. Jagadeesan R. Nayak M. K. Ebert P. R. Daglish G. J. (2021). Gene introgression in assessing fitness costs associated with phosphine resistance in the rusty grain beetle. J. Pest Sci. 94, 1415–1426. 10.1007/s10340-020-01315-6

[B164] Singh S. Chandi A. K. (2019). Physiological influences of pyriproxyfen, a juvenile hormone analogue, on *Bemisia tabaci* (Gennadius). Agric. Res. J. 56 (3), 444–452. 10.5958/2395-146x.2019.00071.1

[B165] Sithanantham S. Ballal C. R. Jalali S. K. Bakthavatsalam N. (Editors) (2013). Biological control of insect pests using egg parasitoids (India: Springer), 15.

[B166] Sparks T. C. Nauen R. (2015). IRAC: mode of action classification and insecticide resistance management. Pestic. Biochem. Phys. 121, 122–128. 10.1016/j.pestbp.2014.11.014 26047120

[B167] Sparks T. C. Dripps J. E. Watson G. B. Paroonagian D. (2012). Resistance and cross-resistance to the spinosyns–a review and analysis. Pestic. Biochem. Phys. 102 (1), 1–10. 10.1016/j.pestbp.2011.11.004

[B168] Steinbach D. Moritz G. Nauen R. (2017). Fitness costs and life table parameters of highly insecticide-resistant strains of *Plutella xylostella* (L.)(Lepidoptera: plutellidae) at different temperatures. Pest Manage Sci. 73 (9), 1789–1797. 10.1002/ps.4597 28444827

[B169] Sun H. Tong K. P. Kasai S. Scott J. G. (2016). Overcoming super‐knock down resistance (super‐kdr) mediated resistance: multi‐halogenated benzyl pyrethroids are more toxic to super‐kdr than kdr house flies. Insect Mol. Biol. 25 (2), 126–137. 10.1111/imb.12206 26691197

[B170] Tabebordbar F. Shishehbor P. Ziaee M. Sohrabi F. (2020). Lethal and sublethal effects of two new insecticides spirotetramat and flupyradifurone in comparison to conventional insecticide deltamethrin on *Trichogramma evanescens* (Hymenoptera: trichogrammatidae). J. Asia Pac Entomol. 23 (4), 1114–1119. 10.1016/j.aspen.2020.09.008

[B171] Tanda A. S. Kumar M. Tamta A. K. Deeksha M. G. (2022). “Advances in integrated management technology of insect pests of stored grain,” in Advances in integrated pest management technology (Cham: Springer), 157–196.

[B172] Uesugi R. (2021). Historical changes in the lethal effects of insecticides against the diamondback moth, *Plutella xylostella* (L.). Pest Manag. Sci. 77 (7), 3116–3125. 10.1002/ps.6344 33639038

[B173] Ullah F. Gul H. Tariq K. Desneux N. Gao X. Song D. (2020a). Functional analysis of cytochrome P450 genes linked with acetamiprid resistance in melon aphid, *Aphis gossypii* . Pestic. Biochem. Phys. 170, 104687. 10.1016/j.pestbp.2020.104687 32980055

[B174] Ullah F. Gul H. Tariq K. Desneux N. Gao X. Song D. (2020b). Fitness costs in clothianidin-resistant population of the melon aphid, *Aphis gossypii* . Plos One 15 (9), e0238707. 10.1371/journal.pone.0238707 32925934 PMC7489515

[B175] Ullah F. Gul H. Desneux N. Said F. Gao X. Song D. (2020c). Fitness costs in chlorfenapyr-resistant populations of the chive maggot, *Bradysia odoriphaga* . Ecotoxicology 29, 407–416. 10.1007/s10646-020-02183-7 32193759

[B176] Ullah F. Gul H. Tariq K. Desneux N. Gao X. Song D. (2021). Acetamiprid resistance and fitness costs of melon aphid, *Aphis gossypii*: an age-stage, two-sex life table study. Pestic. Biochem. Phys. 171, 104729. 10.1016/j.pestbp.2020.104729 33357551

[B177] Ullah F. Xu X. Gul H. Güncan A. Hafeez M. Gao X. (2022). Impact of imidacloprid resistance on the demographic traits and expressions of associated genes in *Aphis gossypii* Glover. Toxics 10(11), 658. 10.3390/toxics10110658 36355949 PMC9696316

[B178] Valmorbida I. Coates B. S. Hodgson E. W. Ryan M. O’Neal M. E. (2022). Evidence of enhanced reproductive performance and lack‐of‐fitness costs among soybean aphids, *Aphis glycines*, with varying levels of pyrethroid resistance. Pest Manag. Sci. 78 (5), 2000–2010. 10.1002/ps.6820 35102702 PMC9310592

[B179] Van Leeuwen T. Dermauw W. Mavridis K. Vontas J. (2020). Significance and interpretation of molecular diagnostics for insecticide resistance management of agricultural pests. Curr. Opin. Insect Sci. 39, 69–76. 10.1016/j.cois.2020.03.006 32361620

[B180] Verheggen F. Barrès B. Bonafos R. Desneux N. Escobar-Gutiérrez A. J. Gachet E. (2022). Producing sugar beets without neonicotinoids: an evaluation of alternatives for the management of viruses-transmitting aphids. Entomol. Gen. 42 (4), 491–498. 10.1127/entomologia/2022/1511

[B181] Vézilier J. Nicot A. De Lorgeril J. Gandon S. Rivero A. (2013). The impact of insecticide resistance on *Culex pipiens* immunity. Evol. Appl. 6 (3), 497–509. 10.1111/eva.12037 23745141 PMC3673477

[B182] Von Santos V. R. Pereira E. J. Guedes R. N. Oliveira M. G. (2013). Does cypermethrin affect enzyme activity, respiration rate and walking behavior of the maize weevil (*Sitophilus zeamais*)? Insect Sci. 20 (3), 358–366. 10.1111/j.1744-7917.2012.01529.x 23955887

[B183] Walsh L. E. Schmidt O. Foster S. P. Varis C. Grant J. Malloch G. L. (2022). Evaluating the impact of pyrethroid insecticide resistance on reproductive fitness in *Sitobion avenae* . Ann. Appl. Biol. 180 (3), 361–370. 10.1111/aab.12738

[B184] Wan Y. Zheng X. Xu B. Xie W. Wang S. Zhang Y. (2021). Insecticide resistance increases the vector competence: A case study in *Frankliniella occidentalis* . J. Pest Sci. 94, 83–91. 10.1007/s10340-020-01207-9

[B185] Wang Z. Y. He K. L. Zhang F. Lu X. Babendreier D. (2014). Mass rearing and release of *Trichogramma* for biological control of insect pests of corn in China. Biol. Control 68, 136–144. 10.1016/j.biocontrol.2013.06.015

[B186] Wang C. Eiden A. Cooper R. Zha C. Wang D. (2019a). Effectiveness of building-wide integrated pest management programs for German cockroach and bed bug in a high-rise apartment building. J. Integr. Pest Manag. 10 (1), 33. 10.1093/jipm/pmz031

[B187] Wang Y. Xiang M. Hou Y. Y. Yang X. Dai H. Li J. (2019b). Impact of egg deposition period on the timing of adult emergence in *Trichogramma* parasitoids. Entomol. Gen. 39, 339–346. 10.1127/entomologia/2019/0896

[B188] Wang L. Ma Y. Guo X. Wan P. Liu K. Cong S. (2019c). Pink bollworm resistance to *Bt* toxin Cry1Ac associated with an insertion in cadherin exon 20. Toxins 11 (4), 186. 10.3390/toxins11040186 30925748 PMC6521048

[B189] Wang C. Eiden A. L. Cooper R. Zha C. Wang D. Hamilton R. G. (2020a). Abatement of cockroach allergens by effective cockroach management in apartments. J. Allergy Clin. Immunol. Pract. 8 (10), 3608–3609. 10.1016/j.jaip.2020.06.040 32615257

[B190] Wang L. Walter G. H. Furlong M. J. (2020b). Temperature, deltamethrin-resistance status and performance measures of *Plutella xylostella*: complex responses of insects to environmental variables. Ecol. Entomol. 45 (2), 345–354. 10.1111/een.12805

[B191] Wang R. Wang Z. Luo C. Yang G. (2020c). Characterization of pyridalyl resistance in a laboratory-selected strain of *Frankliniella occidentalis* . Pestic. Biochem. Phys. 166, 104564. 10.1016/j.pestbp.2020.104564 32448418

[B192] Wang K. Liu M. Wang Y. Song W. Tang P. (2020d). Identification and functional analysis of cytochrome P450 CYP346 family genes associated with phosphine resistance in *Tribolium castaneum* . Pestic. Biochem. Phys. 168, 104622. 10.1016/j.pestbp.2020.104622 32711762

[B193] Wang P. Li M. J. Bai Q. R. Ali A. Desneux N. Dai H. J. (2021). Performance of *Trichogramma japonicum* as a vector of *Beauveria bassiana* for parasitizing eggs of rice striped stem borer, *Chilo suppressalis* . Entomol. Gen. 41 (2), 147–154. 10.1016/j.canrad.2020.06.030

[B194] Weiss B. L. Maltz M. Aksoy S. (2012). Obligate symbionts activate immune system development in the tsetse fly. J. Immunol. Res. 188 (7), 3395–3403. 10.4049/jimmunol.1103691 PMC3311772 22368278

[B195] Xie L. C. Jin L. H. Lu Y. H. Xu H. X. Zang L. S. Tian J. C. (2022). Resistance of lepidopteran egg parasitoids, *Trichogramma japonicum* and *Trichogramma chilonis*, to insecticides used for control of rice planthoppers. J. Econ. Entomol. 115 (2), 446–454. 10.1093/jee/toab254 35039850

[B196] Yin C. Wang R. Luo C. Zhao K. Wu Q. Wang Z. (2019). Monitoring, cross-resistance, inheritance, and synergism of *Plutella xylostella* (Lepidoptera: plutellidae) resistance to pyridalyl in China. J. Econ. Entomol. 112 (1), 329–334. 10.1093/jee/toy334 30371797

[B197] Zang L. S. Wang S. Zhang F. Desneux N. (2021). Biological control with *Trichogramma* in China: history, present status, and perspectives. Annu. Rev. Entomol. 66, 463–484. 10.1146/annurev-ento-060120-091620 32976724

[B198] Zhang F. Yang R. (2019). Life history and functional capacity of the microbiome are altered in beta-cypermethrin-resistant cockroaches. Int. J. Parasitol. 49 (9), 715–723. 10.1016/j.ijpara.2019.04.006 31269412

[B199] Zhang X. Liao X. Mao K. Yang P. Li D. Alia E. (2017). The role of detoxifying enzymes in field-evolved resistance to nitenpyram in the brown planthopper *Nilaparvata lugens* in China. Crop Prot. 94, 106–114. 10.1016/j.cropro.2016.12.022

[B200] Zhang X. Mao K. Liao X. He B. Jin R. Tang T. (2018a). Fitness cost of nitenpyram resistance in the brown planthopper *Nilaparvata lugens* . J. Pest Sci. 91, 1145–1151. 10.1007/s10340-018-0972-2

[B201] Zhang J. H. Zhang S. Yang Y. X. Zhang Y. X. Liu Z. W. (2018b). New insight into foregut functions of xenobiotic detoxification in the cockroach *Periplaneta americana* . Insect Sci. 25 (6), 978–990. 10.1111/1744-7917.12486 28556457

[B202] Zhang X. Wang H. C. Du W. M. Zang L. S. Ruan C. C. Zhang J. J. (2021). Multi-parasitism: a promising approach to simultaneously produce *Trichogramma chilonis* and *T. dendrolimi* on eggs of *Antheraea pernyi* . Entomol. Gen. 41 (6), 627–636. 10.1038/s41419-021-03917-z

